# Trophic levels of decomposers in the treatment of herbicide wastewater: a mediator of positive interactions among microbial community assembly, function and stability

**DOI:** 10.3389/fmicb.2025.1716396

**Published:** 2025-12-11

**Authors:** Fei Xu, Wenjie Wu, Jun Teng, Xinyi Wei, Jinhui Wang, Zheng Zhao, Mengyu Liu, Hao Wang, Qiang Kong

**Affiliations:** 1 College of Geography and Environment, Shandong Normal University, Jinan, Shandong, China; 2 Key Laboratory of Lake and Watershed Science for Water Security, Nanjing Institute of Geography and Limnology, Chinese Academy of Sciences, Nanjing, Jiangsu, China; 3 University of Chinese Academy of Science, Nanjing, Jiangsu, China; 4 Dongying Institute, Shandong Normal University, Dongying, Shandong, China

**Keywords:** trophic levels of decomposers, herbicide wastewater, microbial community assembly, function and stability, microbial diversity

## Abstract

Current wastewater treatment methods tend to acclimate to sludge, but they may not be able to address the presence of herbicides with complex components in agricultural runoff. In this study, we constructed decomposer trophic levels by setting single-channel and multichannel sequencing processes for different herbicide-containing wastewater treatments. Each treatment unit was divided into bacteria-only, wetland plant-only, and wetland plant-microbe continuum treatments. We designed three experiments to investigate the effects of herbicide type, trophic level and biological interaction on system stability, which was predominantly controlled by microbial community assembly processes and functions. The results revealed a threshold for the transition from stochastic to deterministic processes as the concentrations of the herbicides glyphosate (PMG) and atrazine (ATZ) increased. Compared with the effluent water quality of the single herbicide treatment, the decomposer trophic level significantly increased the stochastic effect of the system on effluent water quality. The similarity differences caused by the drift from the parallel units in the primary levels (ca. D_intralevels_ = 0.22) bridged the gap to the secondary levels (ca. D_intralevels_/D_interlevels_ = 1.13), which resulted in the best community stability. Analysis of the microbial community life history strategies indicated that multichannel configurations led to a transition in microbial metabolic capacity (RS selection) and environmental responsiveness (RC selection) under herbicide stress to maintain community stability. Therefore, the system stability in the water treatment process could be optimized by the systematic design of the microbial decomposition trophic level, which is considered an important contributor to the positive coordination between biodiversity and function.

## 1 Introduction

The extensive application of herbicides has resulted in elevated concentrations of herbicide residues (0.1–107 mg/L) and excessive nitrogen and phosphorus loads in agricultural runoff, posing significant threats to ecosystem health and microbial community dynamics (Saleh et al., 2020; Yu et al., 2023). Glyphosate (N-phosphonomethyl glycine; PMG) and atrazine (2-chloro-4-ethylamino-6-isopropylamino-s-triazine; ATZ), two major herbicides containing organophosphorus and nitrogen compounds, respectively, have remained the most commonly used herbicides globally since 2001 (Maggi et al., 2020; Sharma et al., 2019). While these compounds differ in their functions, both exhibit considerable potential risks to human health and the ecosystem because of their persistent residues and pronounced bioenrichment potential in the environment (Jenkins et al., 2017; Mahajan et al., 2020).

Existing herbicide wastewater treatment methods include physical, chemical, and biological methods. Among these methods, biological treatments have received considerable attention because of their relatively high efficiency and low degree of secondary pollution (Saleh et al., 2020). Constructed wetlands (CWs) have advantages in terms of cost-effectiveness and manageability in ameliorating extensively diffused herbicide pollution (Vymazal and Březinová, 2015; Wu et al., 2023). However, the complex chemical characteristics of herbicides often limit the effectiveness of standalone CW systems (Gupta S. et al., 2021). Microbial fuel cells (MFCs), as an emerging technology, enhance the bioelectrochemical processes that accompany wastewater treatment, thereby enhancing the combination of CW and MFC technologies. Compared with individual CW or MFC systems, the CW-MFC system significantly enhances contaminant removal efficiency by improving the interaction effects between plants and microorganisms. Mu et al. (2024) further revealed that exudates from plant roots serve as electron donors to promote electrochemical reactions and pollutant removal efficiency through microbial oxidation and decomposition. Xu et al. (2023) reported that the compensatory effect of microbes can be enhanced by plant-microbe synergy to achieve superior degradation at relatively high herbicide concentrations. However, as the operating time of the cycle increases, the microbial community structure of CW-MFC gradually becomes monotonous, and the insufficient functional stability limits the further improvement of the wastewater treatment efficiency.

Biological water treatment is focused on the composition and function of microorganisms. Goldford et al. (2018) noted that microbial community functions are highly predictable under natural conditions, as they tend to converge to similar structures even under different starting conditions. However, Foster and Bell (2012) cautioned that interspecies competition among similar functional microbes can increase negative consumption. Therefore, a certain degree of niche separation between strains is more conducive to the stable operation of the system (Godoy et al., 2020). Ruan et al. (2024) used the Super Community Combinations (SuperCC) model to construct synthetic microbial consortia with enhanced bioremediation capabilities. Although these artificial systems demonstrate potential advantages for specific tasks, their stability under simplified assembly and subsequent succession processes requires further validation.

The biological processes used in wastewater treatment currently are typically functional under relatively stable influent water conditions, and they primarily target pollutants associated with factors such as chemical oxygen demand (COD), nitrogen, and phosphorus. On one hand, bacteria, such as ammonia-oxidizing bacteria (AOB) and phosphate-accumulating organisms (PAOs), can be manually selected for bioaugmentation for pollution remediation (Nwankwegu et al., 2022). On the other hand, functional partitioning at different stages of wastewater treatment increases the proportion of nonfunctional invaders, e.g., certain pathogens (Hou et al., 2025). These findings suggest that treatment systems that rely on bacterial domestication and series-mode configurations may lack adaptability and stability for managing complex wastewater systems. The process of treating pollutants with microorganisms can be compared to an ecological process involving a decrease in nutrient levels; this process is similar to that performed by organisms at the predator trophic level in macroscale ecology (García-Oliva and Wirtz, 2025). However, few scholars have explored the relationships at the decomposer trophic levels because multiple processes of decomposition may occur simultaneously in natural environments, and trophic levels cannot be clearly defined. Fortunately, biological water treatments exhibit seminatural characteristics. The decomposition order of pollutants in the water treatment process is related to the different process stages mediated by water flow. Based on this, the microbial community can be divided into ordered levels (such as primary and secondary), not based on the predator–prey relationship. This process may follow the principles of the macroscale trophic level (Chen et al., 2021) and the influence of ecological redundancy on the stability of niches (Liang et al., 2025). Therefore, constructing the trophic levels of decomposers in wastewater treatment systems may be useful for enhancing stability and optimizing performance to address the complexity of water treatment.

In this study, we established devices that were divided into the bacteria-only, plant-only, and wetland plant-bacteria continuum treatments on the basis of the CW-MFC. We also constructed the decomposer trophic levels by single-channel and multichannel influent pathways to simulate the levels between the primary and secondary decomposers. The systems are treated with different herbicides to clarify the effects of herbicide type, trophic level, and biological interaction on system stability, which is predominantly controlled by microbial community assembly processes and functions.

## 2 Materials and methods

### 2.1 Experimental device construction and system operation

**Figure 1:**
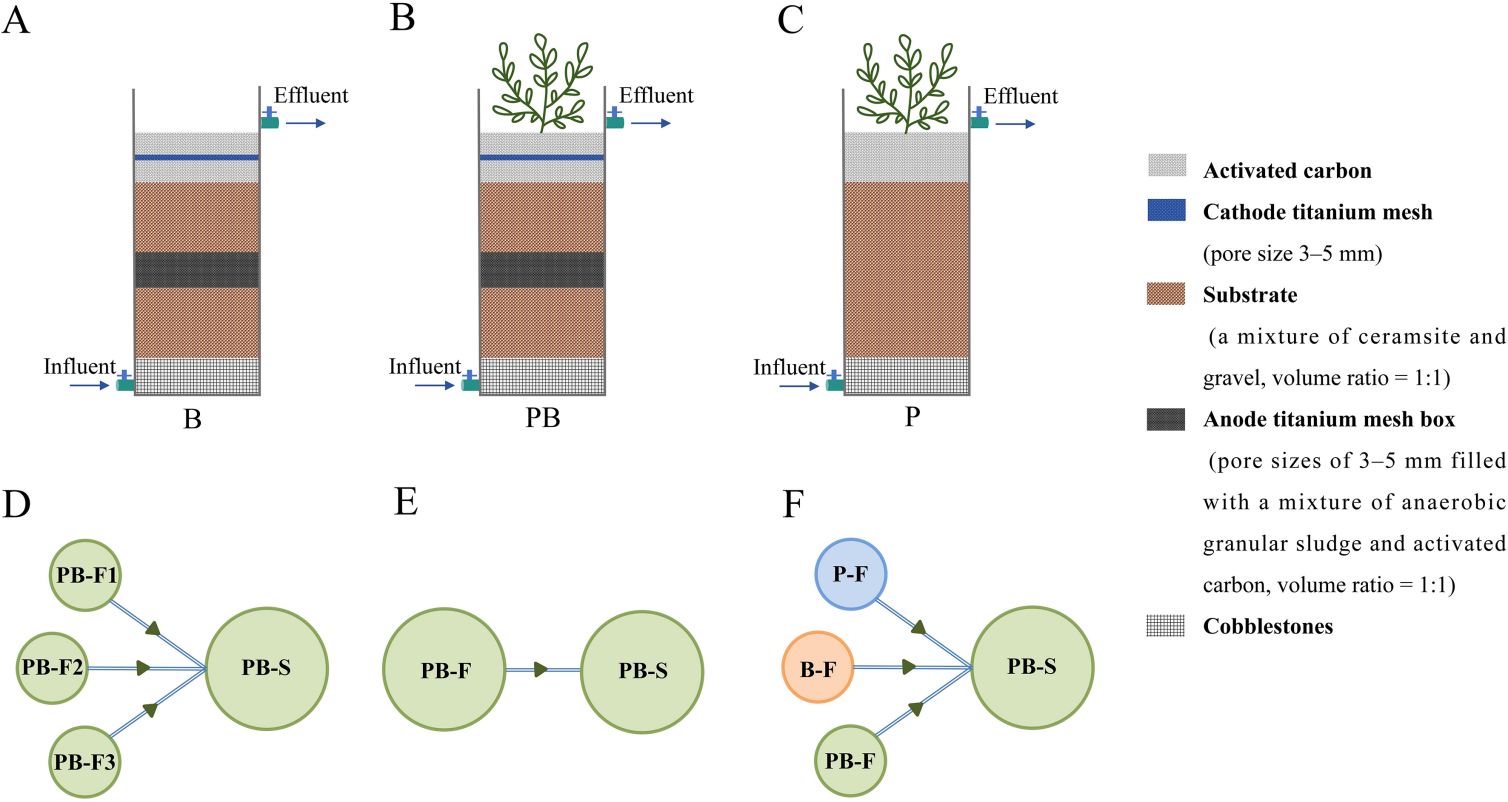
The schematic diagram of the B **(A)**, PB **(B),** and P **(C)** device. Device connection mode of the two-level multichannel PB system **(D)** used in experiment 2, the two-level single-channel PB system **(E)** used in experiment 2, and the two-level multichannel coupled system **(F)** used in experiment 3. The single-channels of the B system and PB system without the secondary level were used in experiment 1. The two levels represent the trophic interlevels, and the parallel units in the primary levels represent the trophic intralevels. The principles for constructing the trophic level were followed: (1) There are differences in biological traits at the same trophic level, and (2) the number of trophic levels gradually decreases. B: Systems with a bacteria-only community; P: Systems with a wetland plant; PB: Systems with a wetland plant-bacterial continuum; F: Primary level; S: Secondary level.

We conducted three different experiments: (1) The first experiment involved studying the impacts of two herbicides on single-channel systems across varying concentration gradients. Single-level and single-channel systems with a bacteria-only community (B) and a wetland plant-bacterial continuum (PB) were established. The system was exposed to simulated agricultural wastewater containing different concentrations of PMG (0, 10, 25, 50, or 100 mg/L) or ATZ (0, 5, 10, or 30 mg/L) (Gupta P. et al., 2021; Liang et al., 2020; Moore et al., 2001). The detailed device constructions used were described in our previously published articles and Figures 1A,B (Xu et al., 2023, 2022). (2) The second experiment involved studying the effects of single-channel and multichannel influent design on microbial community assembly. A two-level multichannel PB system and a two-level single-channel PB system were established. The primary-level and secondary-level devices were named PB-F and PB-S, respectively. The device connections are illustrated in Figures 1D,E. To ensure equal water treatment volumes during each cycle, the diameter of PB-S in the multichannel system was three times larger than that of PB-F, with the same height and layer as. The systems were exposed to simulated agricultural wastewater without herbicides to distinguish the effects of trophic level. For more details regarding device construction, refer to Xu et al. (2024) and Figure 1B. To unify the experimentally designed system, the abbreviation of the device name was changed, differentiating this experiment from that in the original article. (3) The third experiment involved studying the effects of a multichannel design on microbial community assembly in mixed herbicide-containing wastewater. Three identical two-level multichannel coupled systems were established (the device specification design was the same as that of experiment 2’s multichannel system). The first level comprised a plant-only system (P-F), a bacteria-only system (B-F), and a PB system (PB-F), whereas the second stage featured a PB system (PB-S) (Figures 1A–C,F). The three systems were exposed to simulated agricultural wastewater containing 50 mg/L PMG, 10 mg/L ATZ (the optimal concentration treatments in experiment 1), and 25 mg/L PMG mixed with 5 mg/L ATZ [PMG + ATZ; the ratio was determined based on a comprehensive assessment of the hazards to plants and animals, environmental persistence, and potential risks of the herbicides (Brovini et al., 2021; Wang, 2016)]. For more details regarding device construction, refer to Xu et al. (2025). The simulated wastewater composition is detailed in Supplementary Table 1.

The experimental operations for Experiments 1 and 3 were divided into three stages. The first stage was the start-up stage of the systems, where herbicide-free simulated agricultural wastewater was fed into each system without plants until the wastewater treatment capacity showed no significant differences among the cycles. The second stage was the plant acclimatization stage, when plants were added to PB devices until the wastewater treatment capacity of the devices showed no significant differences among the cycles. The above two stages were designed to eliminate the effects of differences in plants and microorganisms on the results of the device. The third stage was the herbicide wastewater treatment stage, when the devices were fed herbicide-containing wastewater for treatment. Each experiment lasted more than 80 days. Experiment 2 was divided into two stages. The first stage was the device start-up stage. The second stage was the stable operation stage, and the duration was the same as that of the third stage in Experiments 1 and 3. The experiment duration was 64 days.

### 2.2 Sampling and measurements

During each cycle, water samples were collected from each device and screened through a 0.45-μm filter membrane. The concentrations of total chemical oxygen demand (COD), ammonia nitrogen (NH_4_^+^-N), total nitrogen (TN), and total phosphorus (TP) were determined using the methods of APHA (2012). The Nemerow pollution index (NPI) method was used to evaluate the effluent quality index. The Class V level of China’s Environmental Quality Standard for Surface Water (GB 3838–2002) was used as the limit value. The data are presented in Supplementary Table 2. The concentration of PMG and ATZ was determined by spectrophotometry (Chinese standards GB 20684–2017), and high-performance liquid chromatography (Chinese standards standard HJ 587–2010), respectively. The data are presented in Supplementary Table 3.

After the experiments, sludge samples were collected from the anodes of each device, and the original acclimated sludge was used as the control check (CK) to analyse the microbial characteristics. The V3 and V4 hypervariable regions of 16S rRNA were amplified by polymerase chain reactions (PCRs) using bacterial universal primers. Amplification and sequencing were performed by Personal Biotechnology Co., Ltd. (Shanghai, China), according to the methods of Xu F. et al. (2021). Then, the bacterial 16S rRNA genes were compared using the Greengenes database, and phylogenetic trees were constructed to calculate the abundance of each taxon (Xu Z. et al., 2021).

### 2.3 Microbial community assembly process analysis

In accordance with the approach based on phylogenetic diversity proposed by Stegen et al. (2012), the null model was constructed via the Picante, Ape, Parallel, and Reshape2 programs in R Studio (Zhang Z. et al., 2019), and the *β*-nearest taxon index (*β*NTI) values and Bray-Curtis-based Raup-Crick (RC_bray_) values were calculated. The microbial community assembly was determined according to Stegen et al. (2012), as shown in Supplementary Figure 1.

### 2.4 Data analysis

All experiments were conducted in triplicate, and the results were presented as the mean ± standard deviation (SD). The opposite NPI value was used as the variable of water quality (WQ) for meta-analysis. The influence of different variables on the stochastic process in the microbial community assembly was analysed by using the Meta program in R Studio. The odds ratio (OR) and 95% Confidence interval (CI) were used as effect indicators. When I^2^ ≤ 50% and *p* ≥ 0.1, the heterogeneity was small, and the fixed effects model was adopted for analysis. In contrast, when I^2^ > 50% and *p* < 0.1, the random effects model was used. Principal component analysis (PCA) was performed using Origin 2025 to analyse the microbial metabolic pathways of each system predicted by KEGG. Microbial life history strategies were analysed on the basis of Piton et al. (2023). The Bray-Curtis distances among the devices were calculated using the Vegan program in R Studio. The relationship regions of the Bray-Curtis distances between the intralevels (D_intralevels_) and interlevels (D_interlevels_) were binomially fitted using the sliding window extremum method in MATLAB. Polynomial curves were used in other simple fitting analyses. The cohesion index was used to evaluate the stability levels of the microbial networks. The Psych, Vegan, and Dplyr program packages in R Studio were used to calculate the positive and negative cohesion levels of the microbial network. The stability of the microbial network was calculated as follows (Hernandez et al., 2021; Herren and McMahon, 2017):

$$\text{Network stability}=\frac{|\text{Negative cohesion}|}{\text{Positive cohesion}}$$

At higher values, the systems were more stable. A structural equation model (SEM) was constructed using the Vegan and PiecewiseSEM packages in R Studio. When the Akaike information criterion (AIC) was < 2 and *p* > 0.05, the model fit was considered reliable. Data processing was conducted through SPSS 26, and graphs were drawn by using Origin 2025 and R Studio.

## 3 Results

### 3.1 Microbial community assembly processes

**Figure 2:**
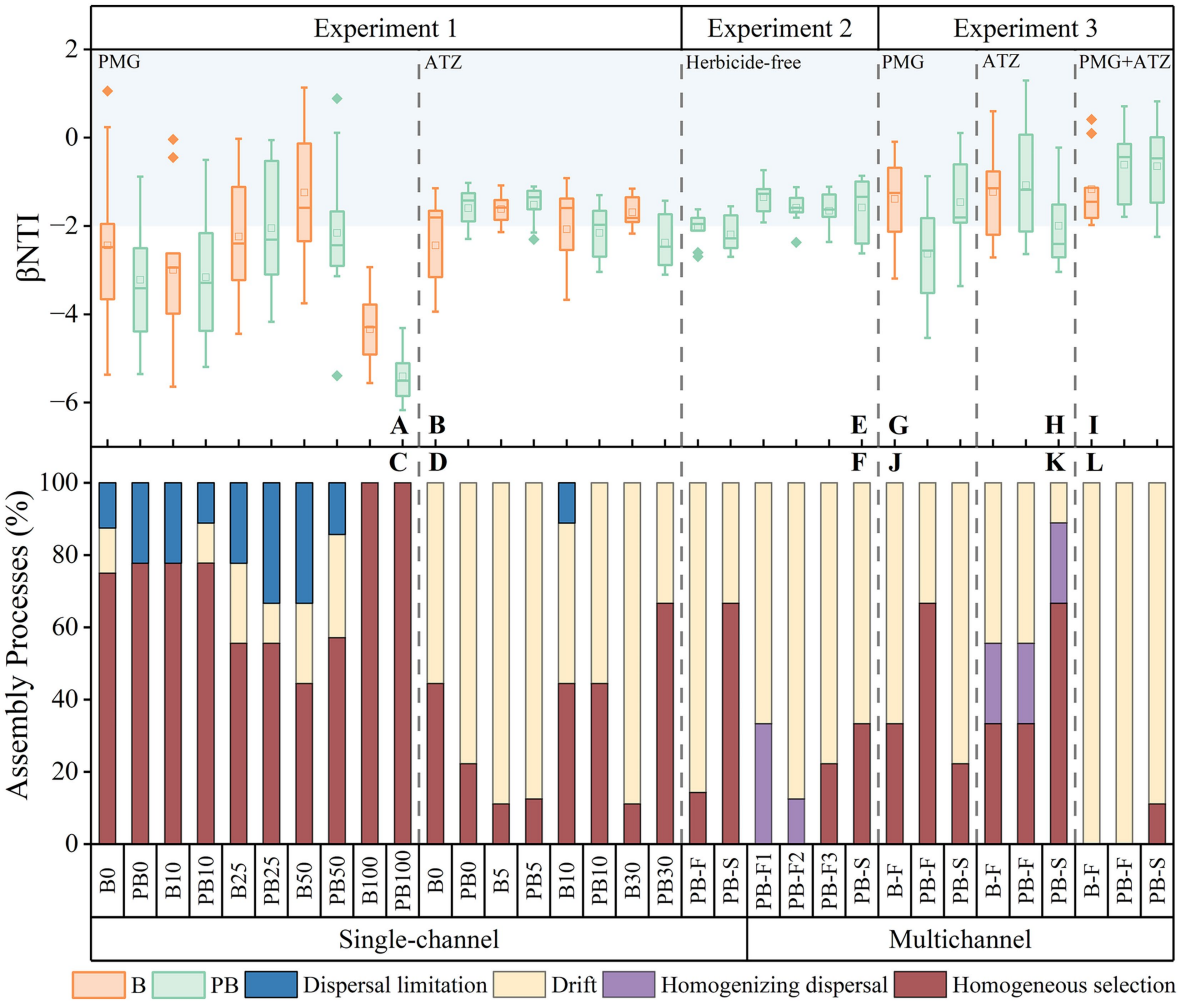
*β*NTI values **(A,B,E,G,H,I)** and community assembly processes **(C,D,F,J,K,L)** of single-channel systems treated with different concentrations of PMG **(A,C)** and ATZ **(B,D)** in experiment 1, a single-channel system and multichannel system treated with herbicide-free **(E,F)** in experiment 2, and the multichannel systems treated with PMG **(G,J)**, ATZ **(H,K)** and PMG + ATZ **(I,L)** in experiment 3. The blue area represents the range of the stochastic process. B: Systems with a bacteria-only community; P: Systems with a wetland plant; PB: Systems with a wetland plant-bacterial continuum; F: Primary level; S: Secondary level. PMG: Glyphosate; ATZ: Atrazine. The numbers after the device (0/5/10/25/30/50/100) correspond to different concentrations of the two pesticides, respectively.

The *β*NTI values and the microbial community assembly processes of different herbicide treatment systems in a single-channel are shown in Figures 2A–D. There was a threshold effect between the stochastic processes and the herbicide concentrations. At medium PMG concentrations and low ATZ concentrations, the stochastic processes showed a peak. A deterministic assembly process dominated by homogeneous selection was subsequently established as the herbicide concentration increased. The proportions of the deterministic process were greater in PMG than in ATZ, which were more similar to those in PB than those in B. The differences were more significant at higher herbicide concentrations.

The *β*NTI values and microbial community assembly processes of each system under different device construction modes are illustrated in Figures 2E,F. The *β*NTI values were greater in the multichannel devices than in the single-channel devices. In the multichannel devices, community assembly in PB-F was a stochastic process dominated by homogenizing dispersal and drift, but only drift occurred under single-channel conditions. The stochastic process of PB-S was weaker than that of PB-F in both single-channel and multichannel devices. However, PB-S in multichannel devices was a stochastic processes dominated by drift (66.7%), whereas PB-S in a single-channel device was a deterministic processes dominated by homogeneous selection (66.7%).

The *β*NTI values and community assembly processes of multichannel systems under different herbicide treatments are presented in Figures 2G–L. Owing to the effects of the herbicides, the stochastic processes in the multichannel systems were slightly weaker compared with those in the herbicide-free systems in experiment 2. However, the overall assembly remained predominantly stochastic. The multichannel system under mixed herbicide stress was characterized by stochastic processes dominated by drift changes, and its stochasticity was significantly greater than that under single herbicide treatments. The microbial community assembly processes at the first level (B-F and PB-F) under the single herbicide treatments were consistent with those under the corresponding herbicide concentrations in experiment 1. The secondary levels differed among the three systems: the stochastic process was stronger in the PMG treatment than in the first level treatment. In contrast, the deterministic process was stronger in the ATZ and mixed treatments.

**Figure 3:**
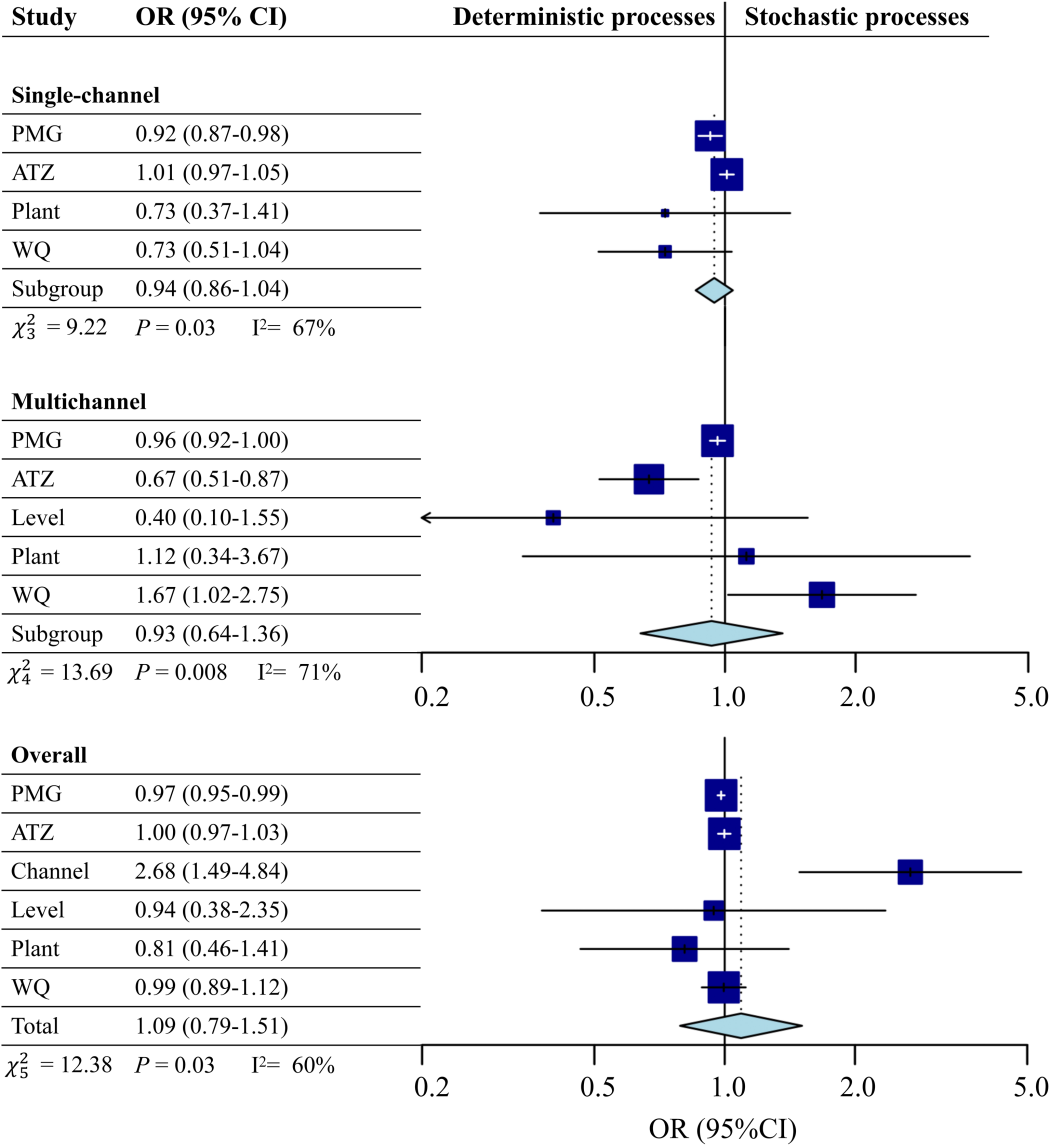
Meta-analysis forest plot of the impacts of different variables on stochastic processes during microbial community assembly processes in systems. A random effects model was used for the meta-analysis. The size of the blue rectangle reflects the weight of each study. The horizontal line represents the point estimate and the 95% confidence interval (CI) of the odds ratio (OR). The diamond at the bottom represents the combined OR value based on the random effects model with its 95% CI. The solid vertical line corresponds to the effect of treatment when the CI includes 1.0. When CI > 1.0, the stochastic process is statistically significant; otherwise, the deterministic process is statistically significant. WQ: Water quality.

A meta-analysis of the impacts of different variables on stochastic processes during microbial community assembly processes is presented in Figure 3. The deterministic processes in the single-channel treatments were dominant and significantly affected by the PMG concentration [OR = 0.92, CI (0.87, 0.98)]. In the multichannel systems, WQ was positively correlated with randomness [OR = 1.67, CI (1.02, 2.75)], and herbicide stress had deterministic effects (OR < 1). Overall, the results indicated that the deterministic processes influenced by herbicides remained. Notably, the probability of stochastic processes under multichannel conditions was 2.68 times greater than that under single-channel conditions [OR = 2.68, CI (1.49, 4.84)], indicating that the multichannel configuration significantly enhanced the stochastic processes of microbial community assembly (*p* < 0.05).

### 3.2 Identification of microbial community functions

**Figure 4:**
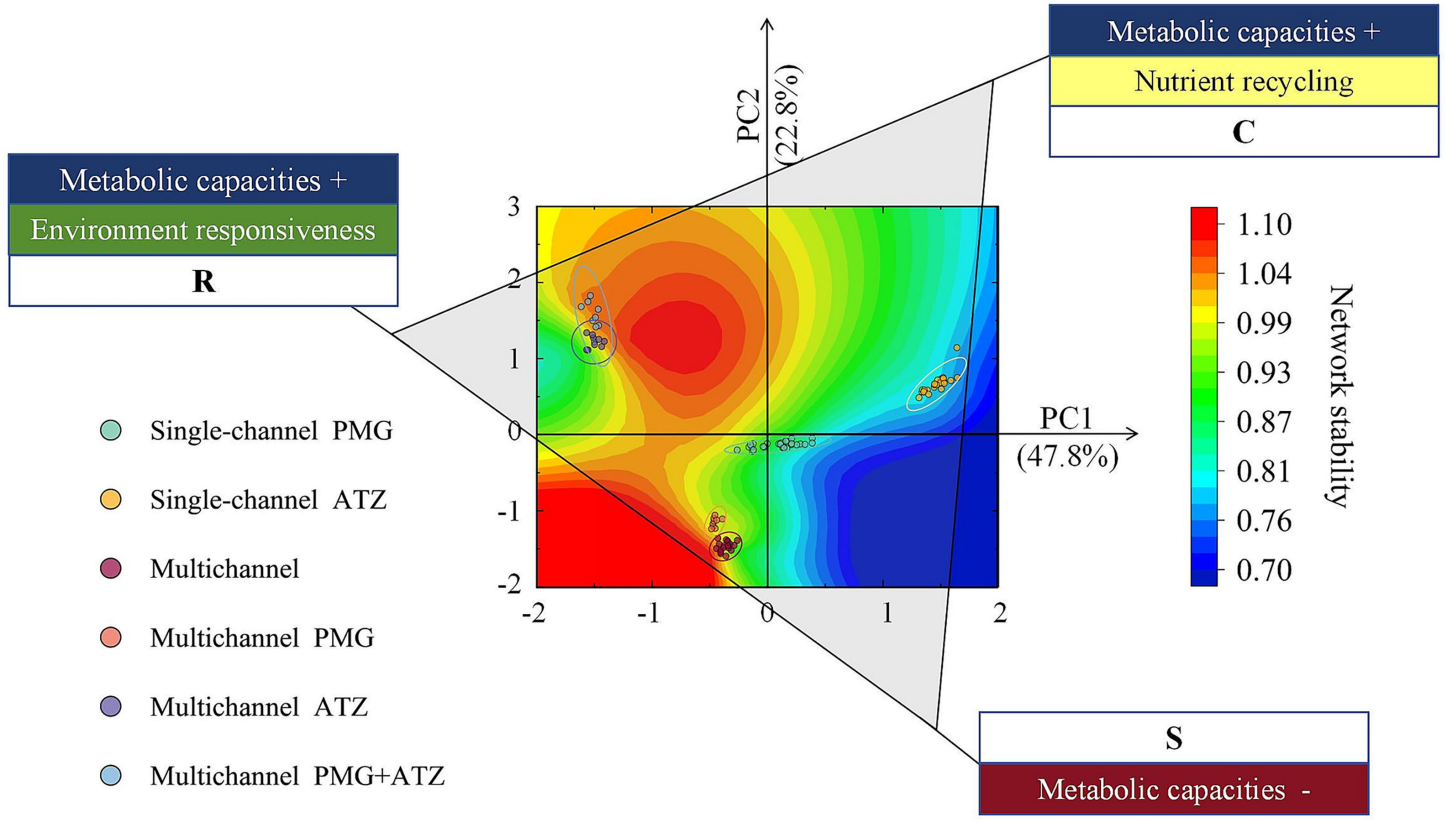
Life history strategies of the microbial communities in each treatment. The scatter location for each system was the same as that in Supplementary Figure 2. The CSR scheme was established by sorting the metabolic pathways of the microbial communities. The functional traits associated with metabolic capacities expanded for nutrient recycling were identified as C-components, those associated with low metabolic capacities were identified as S-components, and those associated with metabolic capacities expanded for environmental responsiveness were identified as R-components. Contour lines reflect the relationship between microbial life history strategies and system network stability. C: Competitor; S: Stress-tolerant; R: Ruderal.

PCA of the microbial metabolic pathways in each system (Supplementary Figure 2) revealed the functional characteristics in each quadrant. The first quadrant was dominated by degradation pathways related to nutrient recovery, including carbohydrate breakdown and amino acid entry into the tricarboxylic acid (TCA) cycle, aligning with the negative direction of the MCOA2 axis described by Piton et al. (2023). In contrast, the second quadrant contained multiple biosynthetic superpathways corresponding to the positive direction of the MCOA1 axis. On the basis of these metabolic axes, we integrated the two-character life history strategy framework proposed in the literature to define the triangular strategy depicted in Figure 4. Fierer (2017) applied the competitor-stress tolerant-ruderal (CSR) strategies proposed for the effects of plants on microorganisms. The multichannel mixed herbicide system and multichannel ATZ system were both of the RC type. The multichannel PMG system and multichannel herbicide-free system were of the RS type. The single-channel ATZ system was of the CS type, and the single-channel PMG system was of the CSR type, which demonstrated weaker competitive effects in the PMG treatment than in the ATZ treatment.

### 3.3 Association analysis of microbial community stability

**Figure 5:**
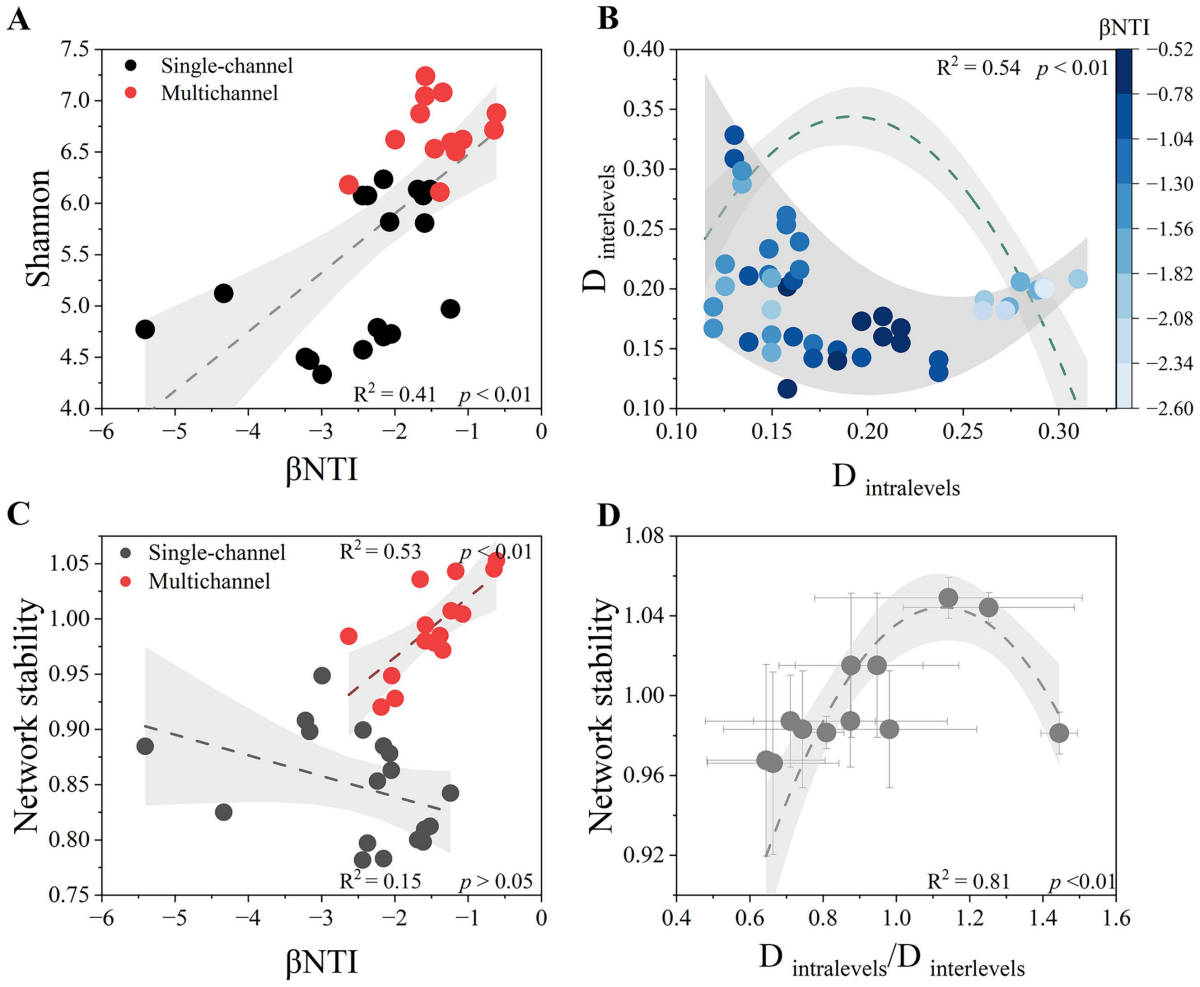
Relationships between the *β*NTI values and Shannon’s diversity index **(A)**. The relationships between the *β*NTI values and Shannon’s diversity index were fitted linearly with 95% confidence intervals. Relationship of the Bray-Curtis distance between the intralevel values (D_intralevels_), interlevel values (D_interlevels_), and *β*NTI values **(B)**. The marginal scatters of the Bray-Curtis distance were fitted by the sliding window extremum method. The relationship between the D_intralevels_ and *β*NTI values was fitted by a polynomial equation, whereas the relationship between the D_interlevels_ and *β*NTI values was not significant and is not presented. Relationships between microbial community assembly processes and system network stability **(C)**. Linear fitting with 95% confidence intervals was used to distinguish the different trends between the single-channel and multichannel devices. Relationships of microbial network stability with D_intralevels_/D_interlevels_ values **(D)**. The changes in microbial network stability were fitted by polynomial with 95% confidence intervals.

The relationships between the *β*NTI value and *α* diversity (Shannon index) are shown in Figure 5A. We found a significant positive correlation between them (*p* < 0.01) in all the treatments. However, the influence of the *β*NTI value on *β* diversity (D_intralevels_ and D_interlevels_) was nonlinear (Figure 5B). The marginal scatters were curve fitted, and the inflection points of the upper and lower lines were connected to determine the central inflection point (D_intralevels_ = 0.22, D_interlevels_ = 0.15). Stronger stochastic processes occurred closer to this point. The formation of this point was influenced mainly by D_intralevels_, as there was a threshold trend for intralevel stochasticity (*p* < 0.01) but not interlevel stochasticity. With increasing D_intralevels_, D_interlevels_ tended to converge, which was accompanied by strengthening of the deterministic process. With decreasing D_intralevels_, D_interlevels_ tended to diverge, resulting in inconsistent community assembly processes. The relationships between *β* diversity and network stability, both intralevel and interlevel, were similar to those between *β* diversity and stochasticity (Supplementary Table 4, Figure 5D), with a tradeoff between the intralevel and interlevel stability (ca. D_intralevels_/D_interlevels_ = 1.13).

Owing to the introduction of trophic levels, a strong positive correlation emerged between stochastic assembly processes and system stability (*p* < 0.01), whereas there was no relationship between them under single-channel conditions (*p* > 0.05) (Figure 5C). The relationships between community functions and system stability varied with different channel designs (Figure 4). Trophic levels enhanced stability through the development of two distinct functions.

**Figure 6:**
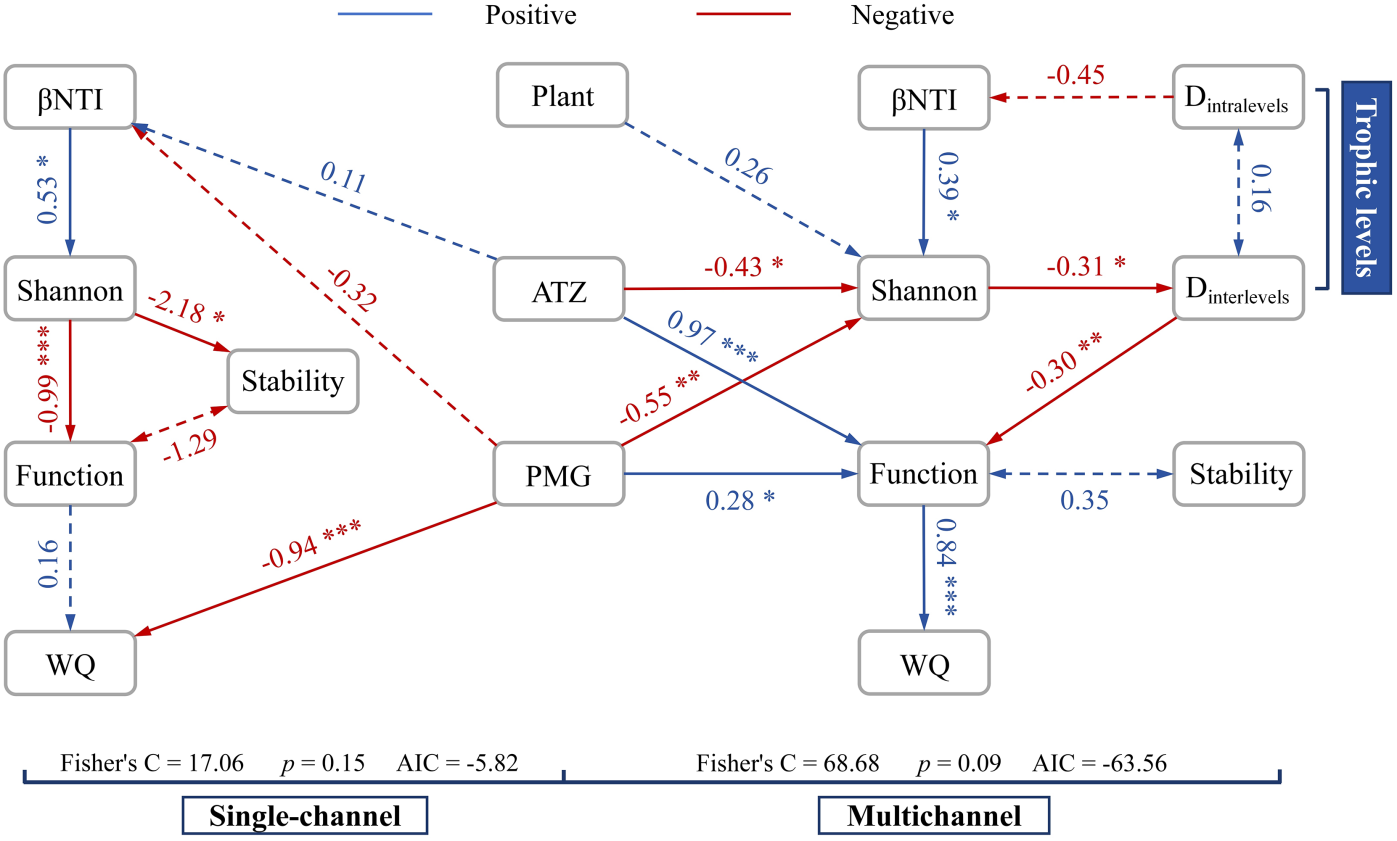
Structural equation modeling (SEM) in single-channel and multichannel systems to analyse the relationships among herbicides (PMG and ATZ), plant effects, microbial diversity (Shannon, D_intralevels_ and D_intralevels_), stochasticity (*β*NTI), functionality, stability, and effluent water quality (WQ) under different device constructions. The solid lines represent significant correlations. The dashed lines represent the nonsignificant correlations in some nonnegligible processes. *p < 0.05, **p < 0.01, ***p < 0.001.

Structural equation modeling (SEM) revealed different process frameworks, such as a chain structure and a network structure, in the single-channel and multichannel systems, respectively (Figure 6). The constructed trophic levels converted the negative effects to positive effects among the diversity, function and stability. The effects of herbicides were more significant and consistent with community diversity and function. Moreover, the positive effects of plants on microbes slightly increased. The effluent water quality was strongly regulated by the positive pathway (*p* < 0.001) in multichannel systems instead of the strong influence of PMG (*p* < 0.001) in the single-channel systems.

## 4 Discussion

### 4.1 Determinism vs. stochasticity

**Figure 7:**
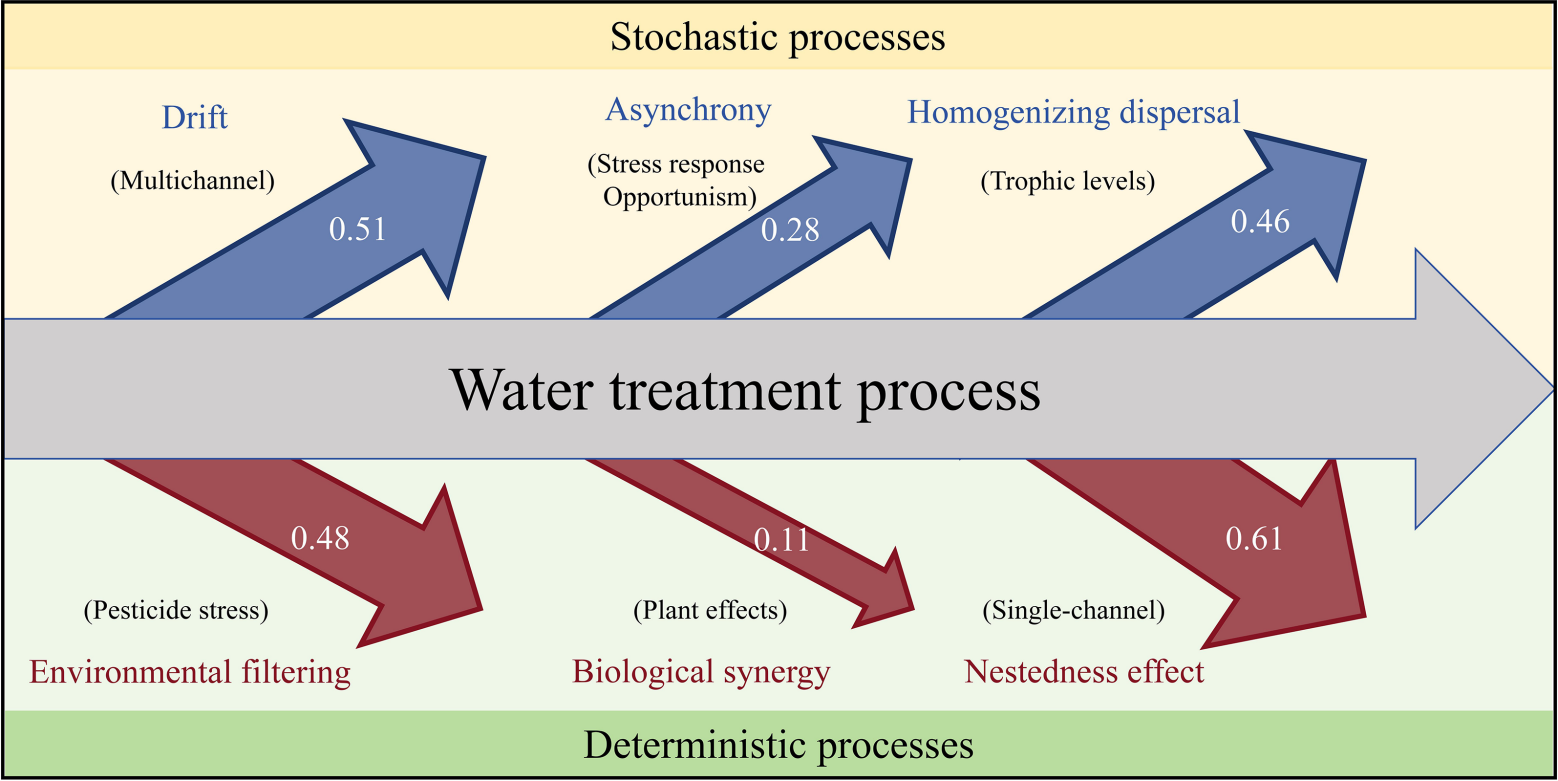
Diagram of the forces influencing the microbial community assembly processes in the water treatment process. The arrow sizes in the figure represent the Δ*β*NTI values of the corresponding influencing factors decomposed through the comparison of different experiments.

In the early stage of community formation, stochasticity plays a dominant role, whereas determinism occurs in the subsequent stage (Dini-Andreote et al., 2015; Yan et al., 2024). This transition mainly occurs during the oriented succession of the natural community when the environment is stable. During water treatment, the complexity and instability of wastewater sources can disturb the natural succession pattern of sludge microorganisms. Therefore, research on community assembly is particularly important. On the basis of our results, we constructed a diagram of the factors influencing microbial community assembly processes during the water treatment process (Figure 7).

In our experiments, herbicide treatment exhibited a deterministic effect on environmental filtering (Figure 3). In particular, when comparing the presence and absence of herbicides in multichannel treatments (Figures 2E–H,J,K), the addition of herbicides significantly reduced the stochasticity of the microbial community assembly processes. The synergistic interactions between plants and microorganisms under stressful conditions has been identified as a deterministic process. The oriented selection of plants is conducive to enriching the beneficial microorganisms and to achieving symbiotic cooperation with microorganisms by recognizing and utilizing the protein signals that they secrete (Xu et al., 2023; Zhao et al., 2024). In single-channel systems, this relationship is obscured by the deterministic nature of herbicides, which is broken down into the enhancement of microbial stochasticity and the deterministic limitation effect of plants on microorganisms (Figures 2A–D, 3, 6). Although a synergistic relationship may exist in the multichannel treatment, the increased environmental heterogeneity from the primary level enhanced the drift effect (Figures 2G,H,J,K). The greater diversity caused by the drift masked the deterministic proportion and increased the advantage of the stochastic processes. In terms of the contribution of plants to environmental heterogeneity, the plant effects in the multichannel treatment showed a weak stochastic processes (Figure 3) and a direct positive correlation with the Shannon diversity instead of with the *β*NTI values (Figure 6).

The intensity of the stress leads to different responses of the microorganisms (Figures 2A–D). Under certain herbicide concentrations, the stress response can be mobilized to enable microbial communities to coordinate and adapt to environmental changes (Mo et al., 2021). At this time, the differences in the timing and mechanism for tolerance adjustment weaken the effect of environmental filtering. The increase in stochasticity is attributed to biological asynchrony (Wagg et al., 2021). High concentrations of herbicides eliminate unsuitable species by homogeneous selection, as the divergence of adaptation differentiation is relatively stable under high-intensity stress (Zhang K. et al., 2019). Therefore, the adaptive responses of microorganisms are the dominant factor in addressing fluctuations in water treatment, which is consistent with the findings in fragile ecological communities (Zhang et al., 2025). The black queen hypothesis suggests that opportunists survive through reductive evolution and by losing public functions under intense stress (Morris et al., 2012). The advantages of increased tolerance and flexible metabolism are acquired from bacterial specialists, which can specifically degrade herbicides and alleviate their toxicity (Ni et al., 2025; Wang X. et al., 2023; Wu et al., 2024). Opportunism can increase the probability of the emergence of bacterial generalists, which positively affects microbial assembly. Herbicide diversity is positively correlated with the abundance of bacterial specialists and opportunists. On the one hand, different specialists exhibit an enhanced capacity to degrade and utilize herbicides and therefore create ecological niches for the coexistence of species (Goldford et al., 2018; Mas et al., 2016; Wang C. et al., 2023). On the other hand, cross-tolerance, which is the response of tolerance to a certain stress to cope with other stresses (also known as cross-protection or cross-response), occurs when microorganisms are exposed to a mixture of toxins (Zhou and Wang, 2023). Therefore, the stochasticity of microorganisms under mixed herbicide treatment is significantly greater than that under single herbicide treatment (Figures 2G–L). The rational utilization of biological stress responses and cross-tolerance establishes a triangular synergistic network involving bacterial specialists, opportunists and toxic substances (Su et al., 2023).

Minor differences in microbial community assembly processes can significantly affect the subsequent development process (Xu et al., 2024). Even if the designs of the primary level devices are similar, the differences in the assembly processes (e.g., drift) among the primary level devices reduce the determinism of nestedness from secondary levels (Figures 2E,F). The construction of trophic levels increases the homogenizing dispersal diversity between neighbouring levels. The diversity of founders at different primary levels enriches the origination of species at the intralevel and mitigates the Matthew effect at the interlevel (Zipple et al., 2025).

### 4.2 *α* diversity vs. *β* diversity

**Figure 8:**
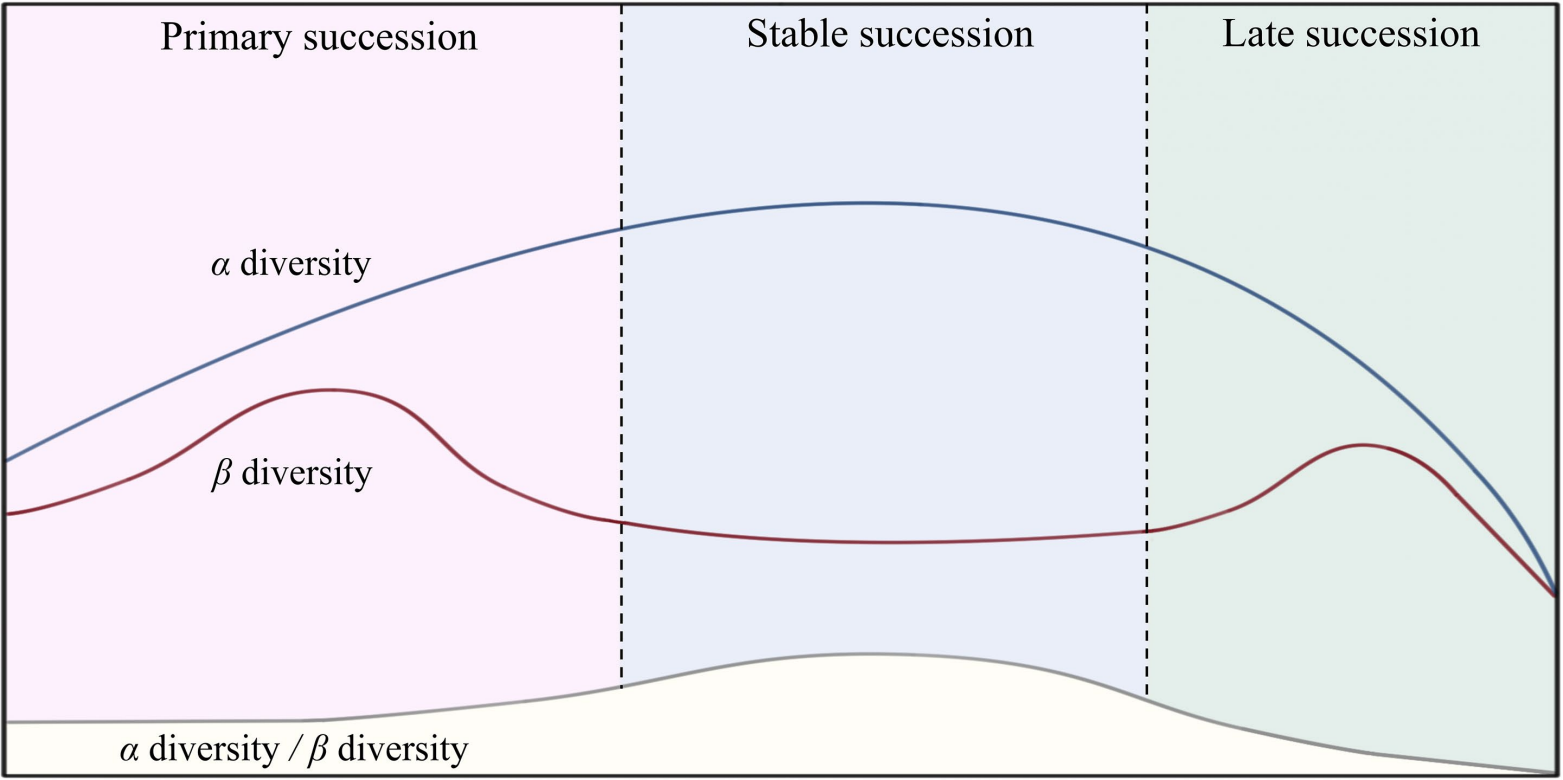
Diagram of the variation patterns of microbial community diversity at different successional stages.

Biodiversity is an indispensable index in community ecology research. Some studies have shown that greater diversity results in a more stable community (Liang et al., 2025), but others have presented the opposite results (Martino et al., 2022; Tilman et al., 2014). This contradiction arises due to the different cognitive perspectives on diversity at distinct spatial and temporal scales. *α*-Diversity, which mainly refers to the number of species in a habitat, is the foundation of biodiversity. Meanwhile, *β* diversity, whose the variation in which is affected by the *α* diversity, refers to the dissimilarity in species composition among different habitat communities (Piazzi and Ceccherelli, 2020). The different spatial scales of *α* and *β* diversity lead to differences in the temporal scales of community succession, especially under conditions of global warming and anthropogenic disturbances (Li et al., 2025; Martino et al., 2022; Wu et al., 2024). In most cases, microbial succession from the pioneer stage to the late stage is characterized by *α* diversity first increasing to stability and then decreasing, along with inconsistent changes in *β* diversity after primary succession (Figure 8). This pattern is consistent with the studies conducted Martino et al. (2022) and Purschke et al. (2013). Therefore, maintaining a proper ratio of *α* to *β* diversity may be a sign of stable community succession.

Traditionally, there is a positive correlation between *α* diversity and the stochastic processes of microbial communities (Xun et al., 2019); that is, determinism is predominant in community assemblies with low diversity, and stochasticity is predominant in those with high diversity. However, we can recognize that in specific cases, such as studies conducted on a small population, an increase in stochasticity can lead to the loss of *α* diversity (Gilbert and Levine, 2017). Our results showed that the *β*NTI values were positively correlated with *α* diversity in all the treatments (Figure 5A), indicating that sludge communities do not have the disadvantages of small populations at this water treatment system scale. The original intention of increasing *α* diversity by enhancing stochastic processes following the conventional ecological theory is feasible.

Therefore, *β* diversity should not have a clear positive correlation with stochastic processes, which is consistent with our results (Figure 5B). The intralevel differences in *β* diversity are generated mainly by turnover, which is generated from an increase in or loss of species caused by drift. The interlevel differences in *β* diversity are caused mainly by nestedness, which reflects the migration diffusion from the primary to secondary levels (Anderson et al., 2011). The sequence mode of conventional wastewater treatment weakens the turnover process between communities, and the gradual increase in nestedness reduces the stability of the systems (Figure 5D). The construction of decomposer trophic levels improves stochasticity by dynamically balancing the intralevel turnover (caused by *α* diversity) and interlevel nestedness (caused by *β* diversity) to quickly achieve stable succession. The values of the threshold in Figures 5B,D all show that the intralevel differences should be greater than the interlevel differences, implying the turnover is more important than nestedness (Martino et al., 2022), and it is consistent with the Kleiber’s law of trophic structure (Perkins et al., 2022), as the average was 0.78 when scaled the threshold values of D_interlevels_ with D_intralevels_.

Therefore, the construction of trophic levels plays an important role in the overall maintenance mechanism of the community, which strongly helps alleviate the negative impact of *α* diversity on the entire system, based on the hypothesis of Gonçalves-Souza et al. (2025). Figure 6 shows that the construction of trophic levels (i.e., the introduction of *β* diversity) positively links *α* diversity with the functions of an ecosystem, which is highly important for the stable operation of a community.

### 4.3 Functions vs. stability

To date, various methods, such as the CSR, Yield-acquisition-stress tolerance (YAS), and competitor-stress tolerator-opportunist (CSO) methods, have been used for the study of microbial functions (Hou et al., 2025; Li et al., 2024; Yang et al., 2023; Zhang et al., 2024). Treseder (2023) reported through comparative research that the CSR model is more suitable for reflecting microbial strategies. In the CSR strategy, microorganisms face a tradeoff among the C, S, and R strategies. C-type microorganisms have a relatively strong ability to acquire resources. S-type microorganisms allocate limited resources to manage the high survival pressure from extreme external environments. R-type microorganisms rapidly grow as opportunists with a narrow ecological niche (Wood et al., 2023). No matter how different the original microbiomes are, they tend to converge to more metabolically active selection (RC in our study in Figure 4) or tolerance selection (RS in our study) (Pascual-García et al., 2025).

The positive effects of biodiversity on ecosystem functions are mediated by complementarity and selection effects, but for ecosystem stability, they are caused by species asynchrony and species stability (Loreau and Hector, 2001). The coordination of ecosystem functions and stability by choosing different combinations of effect mechanisms is an opportunity cost. RC-type microbes like to “travel light” under fluctuating conditions, while RS-type microbes “weather the storm” under stress. Both choices require mutual support between different species. Since there are no universal species that can support all necessary functions simultaneously, functional combinations of species in different environments are needed (Xun et al., 2019).

Ecosystem multifunctionality (EMF) is affected by the seesaw effect: another function may be enhanced when environmental stress leads to a reduction in a specific function (Zhou and Wang, 2023). However, the precondition for the occurrence of this effect is the existence of functional redundancy. Functional redundancy is usually associated with stability in response to disturbances and may be an indirect effect of species diversity (Morales-Salmerón et al., 2025). However, the black queen effect on the stochastic processes in our study is an option for increasing functional redundancy and a mechanism for increasing species diversity (Takeuchi et al., 2024). The relationship between functions and stability may be more closely related to the diversity of functional redundancy rather than to expressive functions. Therefore, the compatibility of multiple functions in the R-strategy may be a key factor affecting stability. Some studies suggest that multiple functions may not simply result from ecological drift; rather, the roles of environmental heterogeneity, dispersal limitation, and differences in the responses and strategies of organisms to the environment should be considered (Louca et al., 2018). Coincidentally, the construction of the trophic levels in our study includes these factors that affect the stochastic processes of the microbial community, positively affecting species diversity, function, and stability (Figure 6).

### 4.4 Inspiration for water treatment

We have summarized the complete evidence chain from community assembly to system performance in Supplementary Table 5. This table demonstrates how environmental pressures and system design, by regulating microbial ecological processes, ultimately affect the processing efficiency of the system. To address the complex requirements of water treatment in the future, some technologies have been developed to improve the compatibility of specialization and generalization in sewage treatment plants (Jin et al., 2023). Biochemical treatments are representative environmentally friendly technologies, e.g., the Bio-integrated Dopp (BioDopp) process, which is based on the concept of enhancing the microbial diversity of sludge to coordinate a variety of biological processes; however, its resistance to fluctuations needs to be strengthened (Shi et al., 2020, 2022). The negative effects among the diversity, functions and stability in single-channel treatments decrease the total effect on water quality. The delayed response to biological regulation has made water quality susceptible to the direct effects of pollutants. In contrast, the construction of decomposer trophic levels led to the formation of two regulatory subsystems: internal assembly and external efficiency. Their dual receivers, namely, *α* diversity and functions, can respond directly to stresses and improve system fitness. Liu et al. (2025) also demonstrated that by adjusting species diversity or composition, the functions and stable relationships can be changed. We can predict the system’s shock resistance capabilities in advance by monitoring the structural characteristics of the microbial community, and seek the dynamic optimal solution between “processing efficiency” and “operational stability”.

Our findings provide an engineering application design principle—constructing decomposer trophic levels via multi-channel configurations. This design can be scaled up through modular stacking and integration into Existing Infrastructure. It can effectively reduce the risk of collapse and minimize the need for chemical agent addition and frequent maintenance. Therefore, this technology demonstrates significant cost-effectiveness advantages in applications requiring high processing reliability. Although this study has revealed the assembly process of the microbial community, the long-term stability and functional resilience of the constructed community still need to be verified over a multi-year operational period. Moreover, conducting pilot-scale experiments based on real wastewater is crucial for promoting this concept towards engineering applications.

## 5 Conclusion

We demonstrate that the construction of decomposer trophic levels significantly enhances system stochasticity. Environmental heterogeneity and the divergent modes of adaptation strategies provide microorganisms with different evolutionary trajectories, thereby reinforcing the biodiversity of the system. In the traditional ecological theory, relatively high biological diversity maintains relatively high ecosystem multifunctionality, while the functions of the microorganisms used in the water treatment industry are usually enhanced through specialized domestication. This study reveals the mechanism underlying this contradiction, i.e., the tradeoff effect at the decomposer trophic level. Appropriate intralevel compositional differences reduce directional convergence at subsequent levels, increase system stochasticity to increase community stability, and promote microbial differentiation into two distinct functional strategies. Furthermore, the relatively restrictive relationship between diversity and functionality/stability in a single-channel device changes into a positive relationship due to the construction of trophic levels. Therefore, nature-based solutions (e.g., simulations of trophic levels in nature) for artificial ecosystems (e.g., wastewater treatment systems) may be preferable options when addressing climate change in the future.

## Data Availability

The original contributions presented in the study are included in the article/Supplementary material, further inquiries can be directed to the corresponding author.

## References

[ref1] Anderson M. J. Crist T. O. Chase J. M. Vellend M. Inouye B. D. Freestone A. L. . (2011). Navigating the multiple meanings of β diversity: a roadmap for the practicing ecologist. Ecol. Lett. 14, 19–28. doi: 10.1111/j.1461-0248.2010.01552.x, 21070562

[ref2] APHA (2012). Standard methods for the examination of water and wastewater. Washington, DC: American Public Health Association.

[ref3] Brovini E. M. de Deus B. C. T. Vilas-Boas J. A. Quadra G. R. Carvalho L. Mendonça R. F. . (2021). Three-bestseller pesticides in Brazil: freshwater concentrations and potential environmental risks. Sci. Total Environ. 771:144754. doi: 10.1016/j.scitotenv.2020.144754, 33736156

[ref4] Chen Y. J. Leung P. M. Wood J. L. Bay S. K. Hugenholtz P. Kessler A. J. . (2021). Metabolic flexibility allows bacterial habitat generalists to become dominant in a frequently disturbed ecosystem. ISME J. 15, 2986–3004. doi: 10.1038/s41396-021-00988-w, 33941890 PMC8443593

[ref5] Dini-Andreote F. Stegen J. C. van Elsas J. D. Salles J. F. (2015). Disentangling mechanisms that mediate the balance between stochastic and deterministic processes in microbial succession. P. Natl. A. Sci. 112, E1326–E1332. doi: 10.1073/pnas.1414261112 PMC4371938 25733885

[ref6] Fierer N. (2017). Embracing the unknown: disentangling the complexities of the soil microbiome. Nat. Rev. Microbiol. 15, 579–590. doi: 10.1038/nrmicro.2017.87, 28824177

[ref7] Foster K. R. Bell T. (2012). Competition, not cooperation, dominates interactions among culturable microbial species. Curr. Biol. 22, 1845–1850. doi: 10.1016/j.cub.2012.08.005, 22959348

[ref8] García-Oliva O. Wirtz K. (2025). The complex structure of aquatic food webs emerges from a few assembly rules. Nat. Ecol. Evol. 9, 576–588. doi: 10.1038/s41559-025-02647-1, 40021903 PMC11976281

[ref9] Gilbert B. Levine J. M. (2017). Ecological drift and the distribution of species diversity. Proc. R. Soc. B 284:20170507. doi: 10.1098/rspb.2017.0507, 28566486 PMC5454268

[ref10] Godoy O. Gómez-Aparicio L. Matías L. Pérez-Ramos I. M. Allan E. (2020). An excess of niche differences maximizes ecosystem functioning. Nat. Commun. 11:4180. doi: 10.1038/s41467-020-17960-5, 32826915 PMC7442808

[ref11] Goldford J. E. Lu N. Bajić D. Estrela S. Tikhonov M. Sanchez-Gorostiaga A. . (2018). Emergent simplicity in microbial community assembly. Science 361, 469–474. doi: 10.1126/science.aat1168, 30072533 PMC6405290

[ref12] Gonçalves-Souza T. Chase J. M. Haddad N. M. Vancine M. H. Didham R. K. Melo F. L. P. . (2025). Species turnover does not rescue biodiversity in fragmented landscapes. Nature 640, 702–706. doi: 10.1038/s41586-025-08688-7, 40074894

[ref13] Gupta P. Pandey K. Verma N. (2021). Improved oxygen reduction and simultaneous glyphosate degradation over iron phthalocyanine and reduced graphene oxide–dispersed activated carbon fiber electrodes in a microbial fuel cell. J. Power Sources 514:230592. doi: 10.1016/j.jpowsour.2021.230592

[ref14] Gupta S. Srivastava P. Patil S. A. Yadav A. K. (2021). A comprehensive review on emerging constructed wetland coupled microbial fuel cell technology: potential applications and challenges. Bioresour. Technol. 320:124376. doi: 10.1016/j.biortech.2020.124376, 33242686

[ref15] Hernandez D. J. David A. S. Menges E. S. Searcy C. A. Afkhami M. E. (2021). Environmental stress destabilizes microbial networks. ISME J. 15, 1722–1734. doi: 10.1038/s41396-020-00882-x, 33452480 PMC8163744

[ref16] Herren C. M. McMahon K. D. (2017). Cohesion: a method for quantifying the connectivity of microbial communities. ISME J. 11, 2426–2438. doi: 10.1038/ismej.2017.91, 28731477 PMC5649174

[ref17] Hou X. Zhang L. Zhao Y. Li J. Jiang Z. Wang S. . (2025). Changes in microbial communities across the whole A^2^/O wastewater treatment process and their drivers—reduced community diversity but increased proportion of certain pathogens. Water Res. 268:122790. doi: 10.1016/j.watres.2024.122790, 39561659

[ref18] Jenkins M. Locke M. Reddy K. McChesney D. S. Steinriede R. (2017). Glyphosate applications, glyphosate resistant corn, and tillage on nitrification rates and distribution of nitrifying microbial communities. Soil Sci. Soc. Am. J. 81, 1371–1380. doi: 10.2136/sssaj2017.02.0063

[ref19] Jin L. Sun X. Ren H. Huang H. (2023). Hotspots and trends of biological water treatment based on bibliometric review and patents analysis. J. Environ. Sci. 125, 774–785. doi: 10.1016/j.jes.2022.03.037, 36375959

[ref20] Li X. Li Y. Shen H. Li S. Zhao Z. Xiao J. . (2024). Different responses of individuals, functional groups and plant communities in CSR strategies to nitrogen deposition in high-altitude grasslands. Sci. Total Environ. 953:176051. doi: 10.1016/j.scitotenv.2024.176051, 39241877

[ref21] Li Y. Wang J. Zhang R. Tian D. Pan J. Liu N. . (2025). Soil bacterial β-diversity as a key determinant of belowground productivity in warming alpine ecosystems. Glob. Chang. Biol. 31:e70161. doi: 10.1111/gcb.70161, 40208740

[ref22] Liang Y. Wei D. Hu J. Zhang J. Liu Z. Li A. . (2020). Glyphosate and nutrients removal from simulated agricultural runoff in a pilot pyrrhotite constructed wetland. Water Res. 168:115154. doi: 10.1016/j.watres.2019.115154, 31630020

[ref23] Liang M. Yang Q. Chase J. M. Isbell F. Loreau M. Schmid B. . (2025). Unifying spatial scaling laws of biodiversity and ecosystem stability. Science 387:eadl2373. doi: 10.1126/science.adl2373, 40112067

[ref24] Liu X. Wang M. Liu B. Chen X. An L. Nie Y. . (2025). Keystone taxa mediate the trade-off between microbial community stability and performance in activated sludges. Nature Water 3, 723–733. doi: 10.1038/s44221-025-00451-6

[ref25] Loreau M. Hector A. (2001). Partitioning selection and complementarity in biodiversity experiments. Nature 412, 72–76. doi: 10.1038/35083573, 11452308

[ref26] Louca S. Polz M. F. Mazel F. Albright M. B. N. Huber J. A. O’Connor M. I. . (2018). Function and functional redundancy in microbial systems. Nat. Ecol. Evol. 2, 936–943. doi: 10.1038/s41559-018-0519-1, 29662222

[ref27] Maggi F. La Cecilia D. Tang F. H. M. McBratney A. (2020). The global environmental hazard of glyphosate use. Sci. Total Environ. 717:137167. doi: 10.1016/j.scitotenv.2020.137167, 32209264

[ref28] Mahajan P. G. Dige N. C. Vanjare B. D. Raza H. Hassan M. Seo S. Y. . (2020). Synthesis and biological evaluation of 1,2,4-triazolidine-3-thiones as potent acetylcholinesterase inhibitors: in vitro and in silico analysis through kinetics, chemoinformatics and computational approaches. Mol. Divers. 24, 1185–1203. doi: 10.1007/s11030-019-09983-y, 31396774

[ref29] Martino C. Dilmore A. H. Burcham Z. M. Metcalf J. L. Jeste D. Knight R. (2022). Microbiota succession throughout life from the cradle to the grave. Nat. Rev. Microbiol. 20, 707–720. doi: 10.1038/s41579-022-00768-z, 35906422 PMC12875531

[ref30] Mas A. Jamshidi S. Lagadeuc Y. Eveillard D. Vandenkoornhuyse P. (2016). Beyond the black queen hypothesis. ISME J. 10, 2085–2091. doi: 10.1038/ismej.2016.22, 26953598 PMC4989313

[ref31] Mo Y. Peng F. Gao X. Xiao P. Logares R. Jeppesen E. . (2021). Low shifts in salinity determined assembly processes and network stability of microeukaryotic plankton communities in a subtropical urban reservoir. Microbiome 9:128. doi: 10.1186/s40168-021-01079-w, 34082826 PMC8176698

[ref32] Moore M. T. Bennett E. R. Cooper C. M. Smith S. Shields F. D. Milam C. D. . (2001). Transport and fate of atrazine and lambda-cyhalothrin in an agricultural drainage ditch in the Mississippi Delta, USA. Agric. Ecosyst. Environ. 87, 309–314. doi: 10.1016/S0167-8809(01)00148-7

[ref33] Morales-Salmerón L. Fernández-Boy E. Herrador B. León R. Domínguez M. T. (2025). Does an enhanced microbial diversity promote the resistance of soil multifunctionality against drought events in amended soils? Biol. Fert. Soils 61, 1013–1031. doi: 10.1007/s00374-025-01914-4

[ref34] Morris J. J. Lenski R. E. Zinser E. R. (2012). The black queen hypothesis: evolution of dependencies through adaptive gene loss. MBio 3, e00036–e00012. doi: 10.1128/mBio.00036-12, 22448042 PMC3315703

[ref35] Mu C. Huang K. Wang L. (2024). Constructed wetland coupled microbial fuel cell (CW-MFC) with *Phragmites australis* planted for hexavalent chromium removal and electricity generation. J Water Process Eng 67:106238. doi: 10.1016/j.jwpe.2024.106238

[ref36] Ni B. Xiao L. Lin D. Zhang T. L. Zhang Q. Liu Y. . (2025). Increasing pesticide diversity impairs soil microbial functions. P. Natl. A. Sci. 122:e2419917122. doi: 10.1073/pnas.2419917122, 39786931 PMC11745395

[ref37] Nwankwegu A. S. Zhang L. Xie D. Onwosi C. O. Muhammad W. I. Odoh C. K. . (2022). Bioaugmentation as a green technology for hydrocarbon pollution remediation, problems and prospects. J. Environ. Manage. 304:114313. doi: 10.1016/j.jenvman.2021.114313 34942548

[ref38] Pascual-García A. Rivett D. W. Jones M. L. Bell T. (2025). Replicating community dynamics reveals how initial composition shapes the functional outcomes of bacterial communities. Nat. Commun. 16:3002. doi: 10.1038/s41467-025-57591-2, 40164605 PMC11958796

[ref39] Perkins D. M. Hatton I. A. Gauzens B. Barnes A. D. Ott D. Rosenbaum B. . (2022). Consistent predator-prey biomass scaling in complex food webs. Nat. Commun. 13:4990. doi: 10.1038/s41467-022-32578-5, 36008387 PMC9411528

[ref40] Piazzi L. Ceccherelli G. (2020). Alpha and beta diversity in Mediterranean macroalgal assemblages: relevancy and type of effect of anthropogenic stressors vs natural variability. Mar. Biol. 167:32. doi: 10.1007/s00227-019-3631-0

[ref41] Piton G. Allison S. D. Bahram M. Hildebrand F. Martiny J. B. H. Treseder K. K. . (2023). Life history strategies of soil bacterial communities across global terrestrial biomes. Nat. Microbiol. 8, 2093–2102. doi: 10.1038/s41564-023-01465-0, 37798477

[ref42] Purschke O. Schmid B. C. Sykes M. T. Poschlod P. Michalski S. G. Durka W. . (2013). Contrasting changes in taxonomic, phylogenetic and functional diversity during a long-term succession: insights into assembly processes. J. Ecol. 101, 857–866. doi: 10.1111/1365-2745.12098

[ref43] Ruan Z. Chen K. Cao W. Meng L. Yang B. Xu M. . (2024). Engineering natural microbiomes toward enhanced bioremediation by microbiome modeling. Nat. Commun. 15:4694. doi: 10.1038/s41467-024-49098-z, 38824157 PMC11144243

[ref44] Saleh I. A. Zouari N. Al-Ghouti M. A. (2020). Removal of pesticides from water and wastewater: chemical, physical and biological treatment approaches. Environ. Technol. Inno. 19:101026. doi: 10.1016/j.eti.2020.101026, 41290285

[ref45] Sharma A. Kumar V. Shahzad B. Tanveer M. Sidhu G. P. S. Handa N. . (2019). Worldwide pesticide usage and its impacts on ecosystem. SN Appl. Sci. 1:1446. doi: 10.1007/s42452-019-1485-1

[ref46] Shi J. Huang W. Han H. Xu C. (2020). Review on treatment technology of salt wastewater in coal chemical industry of China. Desalination 493:114640. doi: 10.1016/j.desal.2020.114640

[ref47] Shi J. Wan N. Li L. Li Z. Han H. (2022). Review on treatment technologies of coal gasification wastewater in China. J. Clean. Prod. 333:130166. doi: 10.1016/j.jclepro.2021.130166

[ref48] Stegen J. C. Lin X. Konopka A. E. Fredrickson J. K. (2012). Stochastic and deterministic assembly processes in subsurface microbial communities. ISME J. 6, 1653–1664. doi: 10.1038/ismej.2012.22, 22456445 PMC3498916

[ref49] Su Y. Xu M. Y. Cui Y. Chen R. Z. Xie L. X. Zhang J. X. . (2023). Bacterial quorum sensing orchestrates longitudinal interactions to shape microbiota assembly. Microbiome 11:241. doi: 10.1186/s40168-023-01699-4, 37926838 PMC10626739

[ref50] Takeuchi N. Fullmer M. S. Maddock D. J. Poole A. M. (2024). The constructive black queen hypothesis: new functions can evolve under conditions favouring gene loss. ISME J. 18:wrae011. doi: 10.1093/ismejo/wrae011, 38366199 PMC10942775

[ref51] Tilman D. Isbell F. Cowles J. M. (2014). Biodiversity and ecosystem functioning. Annu. Rev. Ecol. Evol. Syst. 45, 471–493. doi: 10.1146/annurev-ecolsys-120213-091917

[ref52] Treseder K. K. (2023). Ecological strategies of microbes: thinking outside the triangle. J. Ecol. 111, 1832–1843. doi: 10.1111/1365-2745.14115

[ref53] Vymazal J. Březinová T. (2015). The use of constructed wetlands for removal of pesticides from agricultural runoff and drainage: a review. Environ. Int. 75, 11–20. doi: 10.1016/j.envint.2014.10.026, 25461411

[ref54] Wagg C. Hautier Y. Pellkofer S. Banerjee S. Schmid B. van der Heijden M. G. A. (2021). Diversity and asynchrony in soil microbial communities stabilizes ecosystem functioning. eLife 10:e62813. doi: 10.7554/eLife.62813, 33755017 PMC7987343

[ref55] Wang M. (2016). Visualizing pesticide usage in the United States from 1992 to 2009. Environ Plan A 48, 455–457. doi: 10.1177/0308518X15604760

[ref56] Wang X. Wang Z. Liu W. Liu H. Zhang Q. Zeng J. . (2023). Abundant and rare fungal taxa exhibit different patterns of phylogenetic niche conservatism and community assembly across a geographical and environmental gradient. Soil Biol. Biochem. 186:109167. doi: 10.1016/j.soilbio.2023.109167

[ref57] Wang C. Yu Q. Y. Ji N. N. Zheng Y. Taylor J. W. Guo L. D. . (2023). Bacterial genome size and gene functional diversity negatively correlate with taxonomic diversity along a pH gradient. Nat. Commun. 14:7437. doi: 10.1038/s41467-023-43297-w, 37978289 PMC10656551

[ref58] Wood J. L. Malik A. A. Greening C. Green P. T. McGeoch M. Franks A. E. (2023). Rethinking CSR theory to incorporate microbial metabolic diversity and foraging traits. ISME J. 17, 1793–1797. doi: 10.1038/s41396-023-01486-x, 37596410 PMC10579239

[ref59] Wu H. Wang R. Yan P. Wu S. Chen Z. Zhao Y. . (2023). Constructed wetlands for pollution control. Nat. Rev. Earth Env. 4, 218–234. doi: 10.1038/s43017-023-00395-z

[ref60] Wu M. Xie S. Zang J. Sun Y. Xu S. Li S. . (2024). Multiple anthropogenic environmental stressors structure soil bacterial diversity and community network. Soil Biol. Biochem. 198:109560. doi: 10.1016/j.soilbio.2024.109560

[ref61] Xu F. Liu M. Zhang S. Chen T. Sun J. Wu W. . (2023). Treatment of atrazine-containing wastewater by algae-bacteria consortia: signal transmission and metabolic mechanism. Chemosphere 337:139207. doi: 10.1016/j.chemosphere.2023.139207, 37364639

[ref62] Xu F. Sun R. Wang H. Wang Y. Liu Y. Jin X. . (2021). Improving the outcomes from electroactive constructed wetlands by mixing wastewaters from different beverage-processing industries. Chemosphere 283:131203. doi: 10.1016/j.chemosphere.2021.131203, 34147984

[ref63] Xu F. Wang J. Cai H. Ma F. Lv S. Tian F. . (2025). Using multichannel electrochemical constructed wetlands to treat wastewater containing a mixed herbicide: efficacy and mechanism analysis. J. Environ. Manag. 392:126785. doi: 10.1016/j.jenvman.2025.126785, 40749560

[ref64] Xu F. Wang H. Wei X. Teng J. Wu W. Liu M. . (2024). Ecological processes in separated structures of electroactive wetlands: determinism versus stochasticity. J. Environ. Chem. Eng. 12:113347. doi: 10.1016/j.jece.2024.113347, 41290285

[ref65] Xu Z. Yang Z. Zhu T. Shu W. Geng L. (2021). Ecological improvement of antimony and cadmium contaminated soil by earthworm *Eisenia fetida*: soil enzyme and microorganism diversity. Chemosphere 273:129496. doi: 10.1016/j.chemosphere.2020.129496, 33524758

[ref66] Xu F. Zhao Z. Wang X. Guan W. Liu M. Yu N. . (2022). *Cladophora* can mitigate the shock of glyphosate-containing wastewater on constructed wetlands coupled with microbial fuel cells. Chemosphere 308:136273. doi: 10.1016/j.chemosphere.2022.136273, 36064020

[ref67] Xun W. Li W. Xiong W. Ren Y. Liu Y. Miao Y. . (2019). Diversity-triggered deterministic bacterial assembly constrains community functions. Nat. Commun. 10:3833. doi: 10.1038/s41467-019-11787-5, 31444343 PMC6707308

[ref68] Yan H. Jin X. Zhou X. Gu S. Wu X. Li P. . (2024). Long-term cultivation of grass–legume mixtures changed the assembly process of the microbial community and increased microbial community stability. ISME Commun. 5:ycae157. doi: 10.1093/ismeco/ycae157, 40041708 PMC11879099

[ref69] Yang Y. Dou Y. Wang B. Xue Z. Wang Y. An S. . (2023). Deciphering factors driving soil microbial life-history strategies in restored grasslands. iMeta 2:e66. doi: 10.1002/imt2.66, 38868332 PMC10989924

[ref70] Yu Z. Lu T. Qian H. (2023). Pesticide interference and additional effects on plant microbiomes. Sci. Total Environ. 888:164149. doi: 10.1016/j.scitotenv.2023.164149, 37196943

[ref71] Zhang Z. Deng Y. Feng K. Cai W. Li S. Yin H. . (2019). Deterministic assembly and diversity gradient altered the biofilm community performances of bioreactors. Environ. Sci. Technol. 53, 1315–1324. doi: 10.1021/acs.est.8b06044, 30615833

[ref72] Zhang Y. S. Meiners S. J. Meng Y. Yao Q. Guo K. Guo W. Y. . (2024). Temporal dynamics of grime's CSR strategies in plant communities during 60 years of succession. Ecol. Lett. 27:e14446. doi: 10.1111/ele.14446, 38814284

[ref73] Zhang K. Shi Y. L. Cui X. Yue P. Li K. Liu X. . (2019). Salinity is a key determinant for soil microbial communities in a desert ecosystem. mSystems 4, e00225–e00218. doi: 10.1128/msystems.00225-18, 30801023 PMC6372838

[ref74] Zhang H. Wang B. Wu Y. Wu L. Yue L. Bai Y. . (2025). Plants and soil biota co-regulate stability of ecosystem multifunctionality under multiple environmental changes. Ecology 106:e4534. doi: 10.1002/ecy.4534, 39995294

[ref75] Zhao M. Yang Y. Zhang H. Li Q. Zhao X. Guo X. . (2024). Asymmetric succession in soil microbial communities enhances the competitive advantage of invasive alien plants. Microbiome 12:265. doi: 10.1186/s40168-024-01989-5, 39707566 PMC11662829

[ref76] Zhou L. Wang S. (2023). The bright side of ecological stressors. Trends Ecol. Evol. 38, 568–578. doi: 10.1016/j.tree.2023.01.010, 36906435

[ref77] Zipple M. N. Chang Kuo D. Meng X. Reichard T. M. Guess K. Vogt C. C. . (2025). Competitive social feedback amplifies the role of early life contingency in male mice. Science 387, 81–85. doi: 10.1126/science.adq0579, 39745972 PMC12978263

