# Supplementary material for: Trophic levels of decomposers in the treatment of herbicide wastewater: a mediator of positive interactions among microbial community assembly, function and stability
Source: Front Microbiol. 2025 Dec 11;16:1716396. doi: 10.3389/fmicb.2025.1716396 (PMC12740056; doi:10.3389/fmicb.2025.1716396)
Supplement: Supplementary file 1 [file Data_Sheet_1.zip › Datasheet 1.docx]

**Supplementary material**

**Abbreviations List**

| Abbreviations | Definition | Detail explanation |
| --- | --- | --- |
| AIC | Akaike information criterion | - |
| ATZ | Atrazine | - |
| AOB | Ammonia-oxidizing bacteria | - |
| BioDopp | Bio-integrated Dopp process | - |
| CI | Confidence interval | - |
| CK | Control check | - |
| COD | Chemical oxygen demand | - |
| CWs | Constructed wetlands | - |
| EMF | Ecosystem multifunctionality | - |
| MFCs | Microbial Fuel Cells | - |
| NH_4_^+^-N | Ammonia nitrogen | - |
| OR | Odds ratio | - |
| PMG | Glyphosate | - |
| PCRs | Polymerase chain reactions | - |
| PAOs | Phosphate-accumulating organisms | - |
| SD | Standard deviation | - |
| SEM | Structural equation model | - |
| TCA | Tricarboxylic acid | - |
| TN | Total nitrogen | - |
| TP | Total phosphorus | - |
| SuperCC | Super community combinations | A model used to construct synthetic microbial consortia with enhanced bioremediation capabilities. |
| B-F | Bacteria-only system in the primary level | This is the device for Experiment 3, as shown in Figure 1F. |
| CSO | C-strategy: Competitor  S-strategy: Stress tolerator  O-strategy: Opportunist | A method for studying the life history strategies of microorganisms. |
| CSR | C-strategy: Competitor  S-strategy: Stress tolerant  R-strategy: Ruderal  CS-strategy: Competitor and stress tolerant  RC-strategy: Ruderal and competitor  RS-strategy: Ruderal and stress tolerant | A method for studying the life history strategies of microorganisms. |
| D_intralevels_ | The Bray-Curtis distances between the intralevels | An indicator that quantifies the differences between community samples within a primary level using the Bray-Curtis value. |
| D_interlevels_ | The Bray-Curtis distances between at the interlevels | An indicator that quantifies the differences between community samples at the primary and secondary levels using the Bray-Curtis value. |
| NPI | Nemerow pollution index | A quantitative indicator for water quality assessment, calculated based on data such as COD, NH_4_^+^-N, and TN. |
| P-F | Plant-only system in the primary level | This is the device for Experiment 3, as shown in Figure 1F. |
| PB-F | Wetland plant-bacterial continuum in the primary level | They are the devices for Experiment 2 and 3, as shown in Figure 1D, E, and F. |
| PB-S | Wetland plant-bacterial continuum in the secondary level | They are the devices for Experiment 2and 3, as shown in Figure 1D, E, and F. |
| PCA | Principal component analysis | Used for analyzing the life history strategies of microorganisms. |
| RC_bray_ | Bray-Curtis-Based Raup-Crick | An indicator used to classify the type of a stochastic process, calculated from OTU data. |
| WQ | Water quality | It is the opposite of the NPI value and is used as a variable in meta-analysis and the construction of SEM. |
| YAS | Y-strategy: Yield  A-strategy: Acquisition  S-strategy: stress tolerance | A method for studying the life history strategies of microorganisms. |
| βNTI | β-nearest taxon index | An indicator used to quantify the differences in the phylogenetic structure of a community, calculated based on OTU and phylogenetic tree data. |
| - | Cohesion | A microbial indicator calculated from OTU data can be classified into positive cohesion and negative cohesion.  Positive cohesion: The cooperative relationship between microorganisms Negative cohesion: The competitive relationship among microorganisms |
| - | Function | The indicators for evaluating the life history strategies of microorganisms are derived from the KEGG metabolic pathways. |
| - | Stability | An indicator used to measure the stability of a microbial network, which is calculated based on cohesion. |

**Figure 1.** The partition of the microbial community assembly.

**Figure 2.** PCA analysis of metabolic pathways of microbial communities of each system.

**Table 1** Composition of simulated agricultural farmland surface runoff.

|  | Experiment 1 | |  | Experiment 2 |  | Experiment 3 | | |
| --- | --- | --- | --- | --- | --- | --- | --- | --- |
| Treatment | PMG | ATZ |  | — |  | PMG | ATZ | PMG+ATZ |
| C_12_H_22_O_11_  (mg/L) | 89 | 89 |  | 356.3 |  | 89 | 89 | 89 |
| (NH_4_)_2_SO_4_  (mg/L) | 14.5 | 14.5 |  | 75.4 |  | 14.5 | 14.5 | 14.5 |
| KNO_3_  (mg/L) | 0 | 152.1 |  | 101 |  | 0 | 0 | 0 |
| NaNO_3_  (mg/L) | 128 | 0 |  | 0 |  | 128 | 128 | 128 |
| KH_2_PO_4_  (mg/L) | 8.2 | 8.2 |  | 43.9 |  | 8.2 | 8.2 | 8.2 |
| PMG  (mg/L) | 0/10/25/50/100 | 0 |  | 0 |  | 50 | 0 | 25 |
| ATZ  (mg/L) | 0 | 0/5/10/30 |  | 0 |  | 0 | 10 | 5 |

**Table 2** The effluent concentration and removal efficiency of COD, NH_4_^+^-N, TN, and TP in effluent and NPI of each system.

| Channel | Treatment | Device | COD effluent concentration  (mg/L) ^a^ | COD removal efficiency (%) ^a^ | NH_4_^+^-N effluent concentration  (mg/L) ^a^ | NH_4_^+^-N removal  efficiency  (%) ^a^ | TN effluent concentration  (mg/L) ^a^ | TN removal efficiency  (%) ^a^ | TP effluent concentration (mg/L) ^a^ | TP removal efficiency  (%) ^a^ | NPI ^a^ |
| --- | --- | --- | --- | --- | --- | --- | --- | --- | --- | --- | --- |
| Single- | PMG | B0 | 28.12 ± 0.57 | 69.19 ± 1.34 | 0.42 ± 0.02 | 85.81 ± 1.24 | 4.75 ± 0.24 | 84.81 ± 1.19 | 0.37 ± 0.02 | 80.97 ± 0.97 | 1.84 ± 0.09 |
|  |  | B10 | 29.04 ± 1.36 | 69.76 ± 0.31 | 0.39 ± 0.03 | 86.73 ± 0.74 | 4.12 ± 0.25 | 86.66 ± 1.31 | 0.78 ± 0.04 | 76.44 ± 1.78 | 1.72 ± 0.09 |
|  |  | B25 | 34.07 ± 0.58 | 68.47 ± 0.55 | 0.66 ± 0.04 | 82.08 ± 1.62 | 4.42 ± 0.17 | 86.25 ± 1.08 | 2.75 ± 0.09 | 48.31 ± 2.71 | 5.18 ± 0.17 |
|  |  | B50 | 45.72 ± 1.54 | 66.67 ± 0.63 | 0.76 ± 0.08 | 77.84 ± 1.00 | 5.57 ± 0.12 | 82.88 ± 0.46 | 5.48 ± 0.24 | 34.26 ± 7.01 | 10.19 ± 0.44 |
|  |  | B100 | 59.09 ± 1.52 | 64.87 ± 0.39 | 1.18 ± 0.03 | 72.92 ± 0.76 | 6.79 ± 0.57 | 83.16 ± 0.96 | 9.43 ± 0.30 | 21.80 ± 2.94 | 17.45 ± 0.54 |
|  |  | PB0 | 24.07 ± 0.80 | 75.22 ± 1.37 | 0.31 ± 0.02 | 90.36 ± 0.64 | 4.41 ± 0.17 | 85.09 ± 0.76 | 0.31 ± 0.01 | 84.81 ± 1.04 | 1.69 ± 0.06 |
|  |  | PB10 | 26.30 ± 0.60 | 74.75 ± 1.52 | 0.30 ± 0.02 | 90.70 ± 1.06 | 3.64 ± 0.15 | 88.03 ± 1.30 | 0.68 ± 0.08 | 80.63 ± 2.33 | 1.59 ± 0.09 |
|  |  | PB25 | 34.36 ± 1.01 | 70.14 ± 0.44 | 0.41 ± 0.02 | 87.71 ± 0.78 | 4.74 ± 0.22 | 86.22 ± 0.56 | 2.47 ± 0.07 | 53.35 ± 2.24 | 4.69 ± 0.13 |
|  |  | PB50 | 43.08 ± 1.09 | 68.11 ± 0.29 | 0.74 ± 0.04 | 79.45 ± 0.94 | 5.17 ± 0.19 | 84.69 ± 0.70 | 6.03 ± 0.19 | 30.36 ± 3.46 | 11.18 ± 0.35 |
|  |  | PB100 | 61.14 ± 1.00 | 64.00 ± 0.21 | 1.22 ± 0.03 | 71.61 ± 0.68 | 6.58 ± 0.30 | 82.69 ± 0.45 | 9.70 ± 0.25 | 17.28 ± 2.23 | 17.94 ± 0.46 |
|  | ATZ | B0 | 25.93 ± 2.60 | 74.66 ± 6.27 | 0.09 ± 0.01 | 96.64 ± 0.61 | 1.36 ± 0.17 | 93.03 ± 1.77 | 0.03 ± 0.01 | 98.70 ± 0.54 | 0.59 ± 0.06 |
|  |  | B5 | 29.72 ± 3.42 | 70.96 ± 8.17 | 0.13 ± 0.02 | 94.96 ± 1.63 | 4.50 ± 0.06 | 76.83 ± 0.27 | 0.02 ± 0.01 | 99.44 ± 0.25 | 1.68 ± 0.02 |
|  |  | B10 | 20.74 ± 0.68 | 80.12 ± 0.17 | 0.15 ± 0.02 | 94.06 ± 1.58 | 7.12 ± 0.23 | 63.23 ± 2.10 | 0.03 ± 0.02 | 99.22 ± 0.23 | 2.63 ± 0.09 |
|  |  | B30 | 31.29 ± 3.40 | 69.54 ± 8.01 | 0.19 ± 0.03 | 92.63 ± 1.77 | 5.67 ± 0.17 | 70.73 ± 2.16 | 0.04 ± 0.03 | 98.98 ± 0.40 | 2.11 ± 0.07 |
|  |  | PB0 | 18.09 ± 0.43 | 82.68 ± 0.40 | 0.10 ± 0.02 | 96.06 ± 1.19 | 4.45 ± 0.18 | 77.07 ± 1.72 | 0.02 ± 0.01 | 99.24 ± 0.20 | 1.65 ± 0.07 |
|  |  | PB5 | 15.80 ± 0.85 | 84.74 ± 1.84 | 0.35 ± 0.04 | 86.94 ± 0.61 | 6.07 ± 0.19 | 68.73 ± 1.18 | 0.07 ± 0.04 | 97.86 ± 1.18 | 2.25 ± 0.07 |
|  |  | PB10 | 24.75 ± 0.87 | 76.21 ± 1.58 | 0.09 ± 0.01 | 96.44 ± 0.86 | 2.77 ± 0.20 | 86.78 ± 1.84 | 0.02 ± 0.01 | 99.31 ± 0.26 | 1.05 ± 0.07 |
|  |  | PB30 | 20.58 ± 1.38 | 80.43 ± 1.83 | 0.17 ± 0.03 | 93.52 ± 2.09 | 2.90 ± 0.08 | 85.07 ± 0.56 | 0.03 ± 0.01 | 98.76 ± 0.24 | 1.09 ± 0.03 |
|  | — | PB-F | 32.46 ± 2.87 | 91.48 ± 1.57 | 13.54 ± 1.09 | 3.06 ± 28.32 | 15.38 ± 0.27 | 59.34 ± 2.88 | 5.41 ± 0.07 | 94.57 ± 0.38 | 10.83 ± 0.16 |
|  |  | PB-S | 29.01 ± 0.78 | 92.41 ± 0.38 | 5.94 ± 1.49 | 55.53 ± 23.87 | 9.25 ± 0.35 | 55.25 ± 4.12 | 2.02 ± 0.15 | 82.79 ± 1.45 | 4.50 ± 0.16 |
| Multi- | — | PB-F1 | 37.87 ± 2.75 | 90.05 ± 1.54 | 6.02 ± 1.07 | 57.16 ± 16.18 | 15.38 ± 0.35 | 60.38 ± 1.56 | 2.11 ± 0.05 | 78.54 ± 1.15 | 6.21 ± 0.12 |
|  |  | PB-F2 | 44.40 ± 1.89 | 88.36 ± 1.09 | 6.06 ± 1.26 | 57.32 ± 17.24 | 15.82 ± 0.62 | 59.30 ± 2.61 | 3.29 ± 0.02 | 66.59 ± 0.72 | 6.94 ± 0.08 |
|  |  | PB-F3 | 36.62 ± 4.52 | 90.43 ± 2.35 | 14.27 ± 1.36 | -2.02 ± 30.56 | 17.73 ± 0.49 | 54.27 ± 2.71 | 1.84 ± 0.02 | 81.26 ± 0.53 | 7.51 ± 0.10 |
|  |  | PB-S | 21.82 ± 1.37 | 94.29 ± 0.70 | 1.40 ± 0.51 | 88.27 ± 9.43 | 9.89 ± 0.81 | 74.64 ± 3.68 | 0.61 ± 0.01 | 93.82 ± 0.20 | 3.76 ± 0.28 |
|  | PMG | B-F | 19.31 ± 1.78 | 83.15 ± 2.76 | 0.64 ± 0.05 | 62.35 ± 0.79 | 7.62 ± 0.38 | 62.35 ± 0.79 | 2.73 ± 0.14 | 71.62 ± 0.61 | 5.24 ± 0.25 |
|  |  | PB-F | 14.98 ± 2.02 | 86.98 ± 3.23 | 0.98 ± 0.06 | 64.56 ± 3.56 | 5.15 ± 0.50 | 76.64 ± 0.82 | 2.16 ± 0.11 | 77.59 ± 0.56 | 4.13 ± 0.19 |
|  |  | PB-S | 11.60 ± 0.88 | 89.88 ± 0.92 | 2.10 ± 0.04 | 24.25 ± 2.02 | 5.10 ± 0.18 | 72.33 ± 0.32 | 1.73 ± 0.10 | 82.05 ± 1.42 | 3.38 ± 0.18 |
|  | ATZ | B-F | 15.05 ± 1.31 | 86.35 ± 1.95 | 0.72 ± 0.07 | 73.71 ± 5.72 | 3.75 ± 0.63 | 80.77 ± 6.61 | 0.34 ± 0.02 | 73.73 ± 2.8 | 1.51 ± 0.20 |
|  |  | PB-F | 16.53 ± 0.54 | 84.99 ± 0.4 | 0.76 ± 0.07 | 72.1 ± 3.93 | 7.38 ± 0.41 | 62.46 ± 4.65 | 0.38 ± 0.01 | 70.61 ± 0.53 | 2.78 ± 0.15 |
|  |  | PB-S | 10.67 ± 1.00 | 90.31 ± 0.6 | 0.79 ± 0.04 | 71.09 ± 0.27 | 1.81 ± 0.35 | 90.93 ± 3.32 | 0.21 ± 0.02 | 83.59 ± 3.29 | 0.75 ± 0.12 |
|  | PMG + ATZ | B-F | 12.89 ± 0.81 | 87.60 ± 1.39 | 0.42 ± 0.10 | 85.12 ± 7.40 | 1.69 ± 0.31 | 91.92 ± 2.93 | 0.63 ± 0.06 | 88.67 ± 1.94 | 1.24 ± 0.11 |
|  |  | PB-F | 19.37 ± 1.55 | 81.38 ± 2.46 | 0.57 ± 0.10 | 79.60 ± 7.07 | 6.25 ± 0.05 | 70.31 ± 0.20 | 1.17 ± 0.09 | 78.83 ± 3.30 | 2.65 ± 0.09 |
|  |  | PB-S | 7.52 ± 1.18 | 92.77±2.08 | 0.68 ± 0.10 | 75.46 ± 7.53 | 3.56 ± 0.08 | 83.10 ± 0.19 | 0.30 ± 0.08 | 94.62 ± 2.86 | 1.37 ± 0.03 |

^a^ The results from multiple replicate experiments are reported as mean ± standard deviation (SD).

**Table 3** The effluent concentration and removal efficiency of PMG and ATZ of each system.

| Channel | Treatment | Device | PMG effluent concentration  (mg/L) ^a^ | PMG removal  efficiency (%) ^a^ | ATZ effluent concentration (mg/L) ^a^ | ATZ removal  efficiency (%) ^a^ |
| --- | --- | --- | --- | --- | --- | --- |
| Single- | PMG | B0 | - | - | - | - |
|  |  | B10 | 0.00 ± 0.00 | 100 ± 0.00 | - | - |
|  |  | B25 | 9.99 ± 1.34 | 66.59 ± 3.32 | - | - |
|  |  | B50 | 32.69 ± 2.09 | 39.65 ± 2.24 | - | - |
|  |  | B100 | 60.46 ± 5.76 | 40.28 ± 3.64 | - | - |
|  |  | PB0 | - | - | - | - |
|  |  | PB10 | 0.00 ± 0.00 | 100 ± 0.00 | - | - |
|  |  | PB25 | 9.75 ± 0.60 | 67.11 ± 1.73 | - | - |
|  |  | PB50 | 32.69 ± 1.05 | 39.29 ± 3.93 | - | - |
|  |  | PB100 | 71.32 ± 1.60 | 28.87 ± 4.44 | - | - |
|  | ATZ | B0 | - | - | - | - |
|  |  | B5 | - | - | 0.55 ± 0.08 | 99.89 ± 0.02 |
|  |  | B10 | - | - | 1.58 ± 0.50 | 99.84 ± 0.05 |
|  |  | B30 | - | - | 1.73 ± 0.78 | 99.94 ± 0.03 |
|  |  | PB0 | - | - | - | - |
|  |  | PB5 | - | - | 0.71 ± 0.14 | 99.55 ± 0.28 |
|  |  | PB10 | - | - | 1.42 ± 0.44 | 99.87 ± 0.03 |
|  |  | PB30 | - | - | 2.62 ± 1.57 | 99.91 ± 0.05 |
|  | — | PB-F | - | - | - | - |
|  |  | PB-S | - | - | - | - |
| Multi- | — | PB-F1 | - | - | - | - |
|  |  | PB-F2 | - | - | - | - |
|  |  | PB-F3 | - | - | - | - |
|  |  | PB-S | - | - | - | - |
|  | PMG | B-F | 25.14 ± 2.18 | 36.58 ± 7.06 | - | - |
|  |  | PB-F | 12.29 ± 2.18 | 69.45 ± 4.76 | - | - |
|  |  | PB-S | 8.00 ± 0.83 | 80.04 ± 1.45 | - | - |
|  | ATZ | B-F | - | - | 0.003 ± 0.001 | 99.98 ± 0.01 |
|  |  | PB-F | - | - | 0.002 ± 0.001 | 99.98 ± 0.01 |
|  |  | PB-S | - | - | 0.0005 ± 0.000 | 100.00 ± 0.00 |
|  | PMG + ATZ | B-F | 1.048 ± 0.667 | 94.35 ± 3.40 | 0.001 ± 0.0004 | 99.98 ± 0.01 |
|  |  | PB-F | 1.476 ± 0.407 | 92.06 ± 1.98 | 0.002 ± 0.0002 | 99.96 ± 0.00 |
|  |  | PB-S | 0.000 ± 0.000 | 100.00 ± 0.00 | 0.001 ± 0.0007 | 99.97 ± 0.02 |

^a^ The results from multiple replicate experiments are reported as mean ± standard deviation (SD).

**Table 4** Microbial network stability of each device.

| Single-channel | | |  | Multichannel | | |
| --- | --- | --- | --- | --- | --- | --- |
| Treatment | Device | Network stability ^a^ |  | Treatment | Device | Network stability ^a^ |
| PMG | B0 | 0.899 ± 0.056 |  | — | PB-F1 | 0.972 ± 0.029 |
|  | B10 | 0.949 ± 0.004 |  |  | PB-F2 | 0.980 ± 0.018 |
|  | B25 | 0.853 ± 0.022 |  |  | PB-F3 | 1.036 ± 0.033 |
|  | B50 | 0.842 ± 0.028 |  |  | PB-S | 0.994 ± 0.029 |
|  | B100 | 0.825 ± 0.020 |  | PMG | B-F | 0.985 ± 0.004 |
|  | PB0 | 0.908 ± 0.018 |  |  | PB-F | 0.984 ± 0.011 |
|  | PB10 | 0.898 ± 0.057 |  |  | PB-S | 0.978 ± 0.011 |
|  | PB25 | 0.863 ± 0.023 |  | ATZ | B-F | 1.007 ± 0.023 |
|  | PB50 | 0.885 ± 0.035 |  |  | PB-F | 1.004 ± 0.018 |
|  | PB100 | 0.885 ± 0.024 |  |  | PB-S | 0.928 ± 0.023 |
| ATZ | B0 | 0.782 ± 0.007 |  | PMG + ATZ | B-F | 1.043 ± 0.011 |
|  | B5 | 0.798 ± 0.003 |  |  | PB-F | 1.053 ± 0.014 |
|  | B10 | 0.878 ± 0.130 |  |  | PB-S | 1.045 ± 0.005 |
|  | B30 | 0.800 ± 0.005 |  |  |  |  |
|  | PB0 | 0.810 ± 0.002 |  |  |  |  |
|  | PB5 | 0.812 ± 0.019 |  |  |  |  |
|  | PB10 | 0.783 ± 0.013 |  |  |  |  |
|  | PB30 | 0.797 ± 0.013 |  |  |  |  |
| — | PB-F | 0.949 ± 0.025 |  |  |  |  |
|  | PB-S | 0.920 ± 0.014 |  |  |  |  |

^a^ The results from multiple replicate experiments are reported as mean ± standard deviation (SD).

**Table 5** Summary of the verification of core hypotheses in this study.

|  | Hypothesis | Key Findings | Supporting Evidence | Conclusion |
| --- | --- | --- | --- | --- |
| H1 | An increase in herbicide concentration will drive the microbial community assembly to shift from a stochastic process to a deterministic one. | In the single-channel system, as the concentrations of PMG and ATZ increase, the βNTI value decreases, indicating that the deterministic process dominated by homogenization selection becomes more pronounced. | The βNTI value varies with concentration. (Figure 2A-D) Meta-analysis: The concentration of PMG is negatively correlated with randomness, OR = 0.97. (Figure 3) | Supported |
| H2 | The multichannel trophic levels design can enhance the randomness process and α diversity of the system. | Compared with the single-channel system, the βNTI value of the multichannel system is higher, and the proportion of random processes has significantly increased; moreover, α diversity is positively correlated with βNTI. | Herbicide-free: multichannel vs single-channel. (Figure 2E-F) Meta-analysis: OR for multichannel design is 2.68. (Figure 3) βNTI is positively correlated with Shannon diversity. (Figure 5A) | Supported |
| H3 | The increased randomness and diversity will translate into higher stability of the community network and unique life history strategies. | In a multichannel system, the randomness process is significantly positively correlated with network stability; the system functions are differentiated into strategies such as RS/RC. | In multichannel conditions, βNTI is positively correlated with stability. (Figure 5C) The function strategies are differentiated through PCA analysis. (Figure 4 & Supplementary Figure 2) Network stability data. (Supplementary Table 4) | Supported |
| H4 | The improvement of microbial community characteristics will ultimately enhance the ecosystem function and the quality of the effluent. | The structural equation model indicates that in the multichannel system, randomness and functionality have a direct positive influence on the effluent quality. | Positive correlation paths in the multi-channel SEM model. (Figure 6)  Higher removal rates of PMG and ATZ in the multichannel system. (Supplementary Table 3) | Supported |
| H5 | The balance between intraspecific differences and interspecific differences within a community is crucial for maintaining stability. | There is an optimal β diversity equilibrium point (D_intralevels_ = 0.22, D_intralevels_/D_interlevels_ = 1.13), at which the network stability is at its peak. It is consistent with Kleiber's law of trophic structure. | The nonlinear relationship between β-diversity and stability/randomness as well as the threshold value. ( Figure 5B, D) | Supported |
